# Precise predictions for $V+$jets dark matter backgrounds

**DOI:** 10.1140/epjc/s10052-017-5389-1

**Published:** 2017-12-05

**Authors:** J. M. Lindert, S. Pozzorini, R. Boughezal, J. M. Campbell, A. Denner, S. Dittmaier, A. Gehrmann-De Ridder, T. Gehrmann, N. Glover, A. Huss, S. Kallweit, P. Maierhöfer, M. L. Mangano, T. A. Morgan, A. Mück, F. Petriello, G. P. Salam, M. Schönherr, C. Williams

**Affiliations:** 1 Department of Physics, Institute for Particle Physics Phenomenology, University of Durham, Durham, DH1 3LE UK; 2 Physik-Institut, Universität Zürich, Winterthurerstrasse 190, 8057 Zurich, Switzerland; 3 High Energy Physics Division, Argonne National Laboratory, Argonne, IL 60439 USA; 4 Fermilab, P.O.Box 500, Batavia, IL 60510 USA; 5 Institut für Theoretische Physik und Astrophysik, Universität Würzburg, 97074 Würzburg, Germany; 6 Physikalisches Institut, Albert-Ludwigs-Universität Freiburg, 79104 Freiburg, Germany; 7 Institute for Theoretical Physics, ETH, 8093 Zurich, Switzerland; 8 Theoretical Physics Department, CERN, 1211 Geneva 23, Switzerland; 9 Institut für Theoretische Teilchenphysik und Kosmologie, RWTH Aachen University, 52056 Aachen, Germany; 10 Department of Physics and Astronomy, Northwestern University, Evanston, IL 60208 USA; 11 Department of Physics, University at Buffalo, The State University of New York, Buffalo, 14260 USA

## Abstract

High-energy jets recoiling against missing transverse energy (MET) are powerful probes of dark matter at the LHC. Searches based on large MET signatures require a precise control of the $Z(\nu {\bar{\nu }})+$ jet background in the signal region. This can be achieved by taking accurate data in control regions dominated by $Z(\ell ^+\ell ^-)+$ jet, $W(\ell \nu )+$ jet and $\gamma +$ jet production, and extrapolating to the $Z(\nu {\bar{\nu }})+$ jet background by means of precise theoretical predictions. In this context, recent advances in perturbative calculations open the door to significant sensitivity improvements in dark matter searches. In this spirit, we present a combination of state-of-the-art calculations for all relevant $V+$ jets processes, including throughout NNLO QCD corrections and NLO electroweak corrections supplemented by Sudakov logarithms at two loops. Predictions at parton level are provided together with detailed recommendations for their usage in experimental analyses based on the reweighting of Monte Carlo samples. Particular attention is devoted to the estimate of theoretical uncertainties in the framework of dark matter searches, where subtle aspects such as correlations across different $V+$ jet processes play a key role. The anticipated theoretical uncertainty in the $Z(\nu {\bar{\nu }})+$ jet background is at the few percent level up to the TeV range.

## Introduction

The signature of missing transverse energy (MET) is one of the most powerful tools in the interpretation of data from hadron colliders. In the Standard Model (SM), MET arises from the neutrinos from the decay of *W* and *Z* bosons, and it can be used in their identification and study, as well as in the identification and study of Higgs bosons, top quarks and other SM particles whose decay products include *W* or *Z* bosons. But MET is also an almost omnipresent feature of theories beyond the SM (BSM), where it can be associated to the decay of new particles to *W* and *Z* bosons, or directly to the production of new stable, neutral and weakly interacting particles. Typical examples are theories with dark matter (DM) candidates, or Kaluza–Klein theories with large extra dimensions. Depending on the details, MET is accompanied by other model-discriminating features, such as the presence of a small or large multiplicity of hard jets, or of specific SM particles. The experimental search for these extensions of the SM relies on a proper modeling of the SM backgrounds to the MET signature. The determination of these backgrounds is ideally done by using data control samples, but theoretical input is often helpful, or even necessary, to extend the experimental information from the control to the signal regions, or to extend the application range of the background predictions and to improve their precision [1–3].

In this paper we focus on the theoretical modeling of the SM $V+$ jet backgrounds to inclusive production of large MET recoiling against one or more hadronic jets. These final states address a broad set of BSM models, where the production of an otherwise invisible final state is revealed by the emission of one or more high-$p_\mathrm {T}$ jets from initial-state radiation, where $p_\mathrm {T}$ is the momentum in the transverse plane.^1^ Recent publications by ATLAS [5] and CMS [6, 7], relative to LHC data collected at $\sqrt{s}=13$ TeV, document in detail the current experimental approaches to the background evaluation. The leading background is $Z(\nu {\bar{\nu }})+$ jet production, followed by $W(\ell \nu )+$ jet (in particular for $\ell =\tau$ or when the lepton is outside of the detector).^2^ The experimental constraints on $Z(\nu {\bar{\nu }})+$ jet production at large MET can be obtained from accurate measurements of $V+$ jet production processes with visible vector-boson signatures. It is quite obvious, for example, that the measurement of $Z(\ell ^+\ell ^-)+$ jets with $\ell =e,\mu$ is the most direct and reliable proxy for $Z(\nu {\bar{\nu }})+$ jets. This control sample, however, is statistics limited, due to the smaller branching ratio of *Z* bosons to charged leptons relative to neutrinos. To extrapolate the shape of the *Z* spectrum to the largest $p_\mathrm {T}$ values, therefore, requires a theoretical prediction. The larger statistics of $W(\ell \nu )+$ jets and $\gamma +$ jets events makes it possible to directly access the relevant $p_\mathrm {T}$ range, but the relation between their spectra and the *Z* spectrum needs, once again, theoretical guidance.

**Fig. 1:**
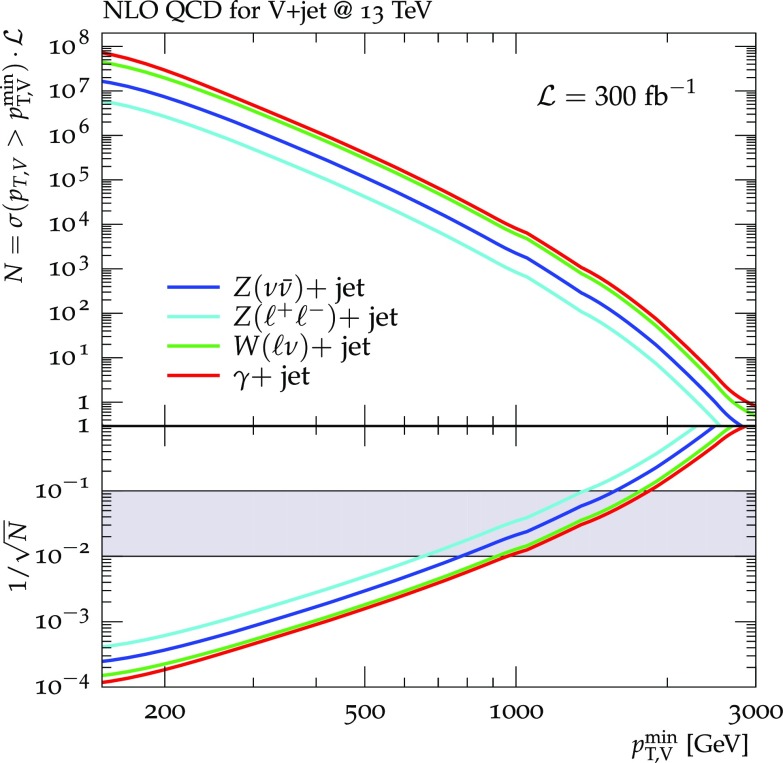
Production rates for $V+$ jet(s), for various decay channels, as a function of the minimum $p_\mathrm {T}$ of the vector boson. Decays into $\ell ^\pm =e^\pm ,\mu ^\pm$ and $\nu _e,\nu _\nu ,\nu _\tau$ are included. The number of events, *N*, is normalized to 300 fb$^{-1}$ of LHC data at $\sqrt{s}=13$ TeV, and includes the basic selection cuts listed in the main body of the paper. The log lower panel shows the statistical uncertainties, calculated as $1/\sqrt{N}$. The gray band in the lower panel indicates the regime of 1–10% statistical uncertainty

To put things into a concrete perspective, Fig. 1 shows the expected event rates, and the relative statistical uncertainty, for 300 fb$^{-1}$ of integrated luminosity at 13 TeV. The extrapolation to the $\mathcal {O}$(100 fb$^{-1}$) and $\mathcal {O}$(3000 fb$^{-1}$) expected from the full run 2 and at the end of the full LHC program, respectively, is straightforward. The $Z(\ell ^+\ell ^-)+$jets data allow for a direct estimate of the $Z(\nu {\bar{\nu }})+$jets rate with a statistical precision below 1% for $p_\mathrm {T}$ up to about 600 GeV. Using the $W(\ell \nu )+$jets or $\gamma +$jets data could in principle extend this range up to about 900 GeV. Beyond this value, the statistical precision of the $W(\ell \nu )+$jets and $\gamma +$jets events remains a factor of 2 better than that of the $Z(\nu {\bar{\nu }})+$jets signal. In order to ensure that the theoretical systematics in the extrapolation from the $W+$jets and $\gamma +$jets rates to the $Z+$jets rates remains negligible with respect to the statistical uncertainty, the former should be kept at the level of a few percent up to $p_\mathrm {T}\sim 2$ TeV, and around 10% up to $p_\mathrm {T}\sim 2.5$–3 TeV, which is the ultimate kinematic reach for the $Z(\nu {\bar{\nu }})+$jets signal at the end of LHC data taking.

The main result of this work is to prove that, thanks to the recent theoretical advances, these goals can be met. This proof requires the analysis of a series of possible effects. On the one hand, the theoretical extrapolation to larger $p_\mathrm {T}$ of the very precise $Z(\ell ^+\ell ^-)+$jets data requires firm control over the shape of the distribution. Several effects, from the choice of parton distribution functions (PDFs) to the choices made for the renormalization and factorization scales used in the calculations, can influence the extrapolation. On the other hand, the level of correlation between the *W*, $\gamma$ and *Z* spectra must be kept under control. At large $p_\mathrm {T}$, in particular, large and process-dependent corrections arise due to the growth of the electroweak (EW) corrections, and these may spoil the correlation induced by pure QCD effects. For our analysis we shall use the most up-to-date theoretical predictions available today for the description of vector-boson production at large $p_\mathrm {T}$. On the QCD side, we rely on the next-to-next-to-leading order (NNLO) calculations, which appeared recently for $Z+$jet [8–12], $W+$jet [13, 14] and $\gamma +$jet [15, 16] production. On the EW side, we apply full NLO calculations for $Z+$jet [17–19], $W+$jet [19, 20] and $\gamma +$jet [21] production with off-shell decays of the *Z* and *W* bosons. Given the strong enhancement of EW Sudakov effects in the TeV region, we also include two-loop logarithmic terms at next-to-leading logarithmic (NLL) accuracy for all $V+$ jet processes [22–25]. An extensive assessment and discussion of the estimates of missing higher-order terms, and of the relative systematics, is given in the main body of this paper. In particular, in order to address non-trivial issues that arise in the context of dark matter searches, we introduce a global framework for the estimate of theoretical uncertainties in all $V+$ jet processes, taking into account correlation effects across different processes and $p_\mathrm {T}$ regions. Also the uncertainties associated with the combination of QCD and EW corrections are discussed in detail.

From the experimental perspective, the determination of the background composition in signal and control regions, and the modeling of other key aspects of experimental analyses (e.g. lepton identification and reconstruction, missing energy, etc.) require a theoretical description of the various $V+$ jets processes at the particle level. Typically, this is provided by Monte Carlo (MC) samples based on multi-jet merging at LO or NLO QCD, and improvements based on higher-order theoretical calculations can be implemented through reweighting of MC events. For the fit of MC predictions to data, ATLAS and CMS analyses rely on the profile likelihood approach, where experimental and theoretical uncertainties are described in terms of nuisance parameters with Gaussian distributions. In this context, the correlations of theoretical uncertainties across $p_\mathrm {T}$ bins (shape uncertainties) and across different $V+$ jets processes play a key role for searches at large MET.

For the implementation of higher-order QCD and EW corrections and for the estimate of theoretical uncertainties in the experimental analysis framework, we propose a procedure based on a one-dimensional reweighting of MC samples. The proposed framework should enable the experiments to carry out their profile likelihood approach, quantifying the impact of the theoretical systematics in their analyses, and validating directly with data the reliability and robustness of the theoretical inputs. In this respect, we would like to stress that, independently of the application to BSM searches, the results in this paper provide a framework for incisive validations of the theoretical calculations. Furthermore, these results might allow for further constraints on PDFs [3, 26].

If the experimental analyses of the MET+jets channel should confirm the usefulness of the approach we propose, the same framework could be adapted to more complex or exclusive final states, in which for example MET is accompanied by a large number of (hard) jets or by specific objects (photons, heavy quarks, Higgs, etc.). These extensions are left for future studies.

The structure of this paper is as follows: In Sect. 2 we introduce the reweighting technique, to incorporate in a MC analysis the effect of higher-order corrections and of their systematic uncertainties including correlations. Section 3 describes details of the setup for our numerical calculations, the employed tools and methods, as well as the detailed definition of physics objects and observables to be used in the context of MC reweighting. In Sect. 4 we discuss higher-order QCD and EW corrections, including the contribution of photon-initiated processes and real vector-boson emission. We present here our approach to the estimate of the various systematics, covering QCD scale, shape and process-dependent uncertainties, as well as uncertainties arising from higher-order EW and mixed QCD–EW corrections. Section 5 contains our summary and conclusions. As detailed in Appendix A, results for all $V+$jets processes are available in form of one-dimensional histograms in the vector-boson $p_\mathrm {T}$ covering central predictions and all mentioned uncertainties. Technical plots on the individual sources of QCD and EW uncertainties are documented in Appendix B.

## Reweighting of Monte Carlo samples

The reweighting of MC samples is an approximate, but straightforward and easy to implement method of combining (N)LO MC simulations with (N)NLO QCD+NLO EW perturbative calculations and to account for the respective uncertainties in a systematic way. The following formula describes the one-dimensional reweighting of MC samples for $V+$ jet production ($V=\gamma ,Z,W^\pm$) in a generic variable *x*,

1 $$\begin{aligned} \frac{\mathrm{d}}{\mathrm{d}x}\frac{\mathrm{d}}{\mathrm{d}{} \mathbf{y}}\,&\sigma ^{(V)}({\varvec{\varepsilon }}_\mathrm {MC},{\mathbf {\varepsilon }}_\mathrm {TH})\nonumber \\&= \frac{\mathrm{d}}{\mathrm{d}x}\frac{\mathrm{d}}{\mathrm{d}{} \mathbf{y}}\sigma ^{(V)}_{\mathrm {MC}}({\varvec{\varepsilon }}_\mathrm {MC}) \left[ \frac{\frac{\mathrm{d}}{\mathrm{d}x}\sigma ^{(V)}_{\mathrm {TH}}({\varvec{\varepsilon }}_\mathrm {TH})}{\frac{\mathrm{d}}{\mathrm{d}x}\sigma ^{(V)}_{\mathrm {MC}}({\varvec{\varepsilon }}_\mathrm {MC})}\right] . \end{aligned}$$

In the case at hand, i.e. $V+$ jet production, the one-dimensional parameter *x* should be understood as the vector-boson transverse momentum, $x=p^{(V)}_\mathrm {T}$, while ${\mathbf {y}}$ generically denotes the remaining variables of the fully differential kinematic dependence of the accompanying QCD and QED activity, including both extra jet and photon radiation, as well as leptons and neutrinos from hadron decays. It is implicitly understood that $\frac{\mathrm{d}}{\mathrm{d}x}\frac{\mathrm{d}}{\mathrm{d}{} \mathbf{y}}\sigma$ depends on *x* and ${\mathbf {y}}$, while in $\frac{\mathrm{d}}{\mathrm{d}x}\sigma$ the variables ${\mathbf {y}}$ are integrated out.

The labels MC and TH in Eq. (1) refer to Monte Carlo and higher-order theoretical predictions, respectively, and the related uncertainties are parametrized through nuisance parameters ${\varvec{\varepsilon }}_\mathrm {TH}, {\mathbf {\varepsilon }}_\mathrm {MC}$. Our recommendations for theory uncertainties in Sect. 4 are formulated in terms of intervals for the related nuisance parameters,

2 $$\begin{aligned} -1<\varepsilon _{\mathrm {TH},k}<1, \end{aligned}$$

which pragmatically should be understood as the $1\sigma$ range of Gaussian uncertainties.

Monte Carlo uncertainties, described by ${\varvec{\varepsilon }}_\mathrm {MC}$, must be correlated in the numerator and denominator on the r.h.s. of Eq. (1), while they can be kept uncorrelated across different processes (apart from $Z(\nu {\bar{\nu }})+\,\mathrm {jet}$ and $Z(\ell ^+\ell ^-)+\,\mathrm {jet}$).

We note that, as opposed to an approach based only on ratios of $p_\mathrm {T}$ distributions, where theory is used for extrapolations across different processes at fixed $p_\mathrm {T}$, MC reweighting is more powerful as it supports all possible extrapolations across different processes and $p_\mathrm {T}$ regions. In particular, it makes it possible to exploit $V+$ jet precision measurements at moderate $p_\mathrm {T}$ in order to constrain $Z(\nu {\bar{\nu }})+\mathrm {jet}$ production in the TeV region.

A further advantage of the reweighting approach (1) lies in the fact that the three terms on the r.h.s. of Eq. (1) do not need to be computed with the same numerical setup (parameters, cuts, observables, etc.). More precisely, only the definition of the variable *x* and the binning of its distribution need to be the same in all three terms. Scale choices, QCD and EW input parameters and PDFs should be the same only in the numerator and denominator of

3 $$\begin{aligned} R_\mathrm {MC}(x,{\mathbf {y}})=\frac{\frac{\mathrm{d}}{\mathrm{d}x}\frac{\mathrm{d}}{\mathrm{d}{} \mathbf{y}}\sigma ^{(V)}_{\mathrm {MC}}}{\frac{\mathrm{d}}{\mathrm{d}x}\sigma ^{(V)}_{\mathrm {MC}}}, \end{aligned}$$

but can be chosen in a different way in $\frac{\mathrm{d}}{\mathrm{d}x}\sigma ^{(V)}_\mathrm {TH}$, provided that QCD and EW corrections themselves are computed using the same settings. Vice versa, possible cuts must be identical only in the numerator and denominator of

4 $$\begin{aligned} R_{\mathrm {TH}/\mathrm {MC}}(x)=\frac{\frac{\mathrm{d}}{\mathrm{d}x}\sigma ^{(V)}_{\mathrm {TH}}}{\frac{\mathrm{d}}{\mathrm{d}x}\sigma ^{(V)}_{\mathrm {MC}}}, \end{aligned}$$

while particle-level MC predictions, $\frac{\mathrm{d}}{\mathrm{d}x}\frac{\mathrm{d}}{\mathrm{d}{} \mathbf{y}}\sigma ^{(V)}_\mathrm {MC}$, can be subject to more exclusive or inclusive cuts in the experimental analysis.

For an optimal combination of higher-order calculations and MC predictions, two conditions should be fulfilled. On the one hand, theory calculations should describe the distribution in the reweighting variable with higher (or at least equal) precision as compared to the MC sample,

5 $$\begin{aligned} \varDelta \left[ \frac{\mathrm{d}}{\mathrm{d}x}\sigma ^{(V)}_{\mathrm {TH}} \right] \le \varDelta \left[ \frac{\mathrm{d}}{\mathrm{d}x}\sigma ^{(V)}_{\mathrm {MC}}\right] . \end{aligned}$$

On the other hand, the MC sample should be more accurate than TH calculations in describing the correlation between *x* and all other variables ${\mathbf {y}}$,

6 $$\begin{aligned} \varDelta \left[ \frac{\frac{\mathrm{d}}{\mathrm{d}x}\frac{\mathrm{d}}{\mathrm{d}{} \mathbf{y}}\sigma ^{(V)}_{\mathrm {MC}}}{\frac{\mathrm{d}}{\mathrm{d}x}\sigma ^{(V)}_{\mathrm {MC}}}\right] \le \varDelta \left[ \frac{\frac{\mathrm{d}}{\mathrm{d}x}\frac{\mathrm{d}}{\mathrm{d}{} \mathbf{y}}\sigma ^{(V)}_{\mathrm {TH}}}{\frac{\mathrm{d}}{\mathrm{d}x}\sigma ^{(V)}_{\mathrm {TH}}}\right] . \end{aligned}$$

More precisely, condition (6) needs to be fulfilled only for those aspects of $V+\,$jet events that are relevant for the actual experimental analysis.

As concerns the first condition, we note that, depending on the choice of the observable *x*, using state-of-the-art theory calculations that involve higher-order QCD and EW corrections may not guarantee that Eq. (5) is fulfilled. In fact, there are a number of aspects, i.e. resolved multi-jet emissions, the resummation of soft logarithms in the region of small vector-boson $p_\mathrm {T}$, soft QCD radiation of non-perturbative origin, multiple photon radiation, or neutrinos and charged leptons resulting from hadron decays, for which fixed-order perturbative calculations of $pp\rightarrow V+$ jet are less accurate than MC simulations.

Thus, the reweighting variable *x* should be defined such as to have minimal sensitivity to the above-mentioned aspects. In this respect, due to its reduced sensitivity to multiple jet emissions, the vector-boson $p_\mathrm {T}$ is a natural choice. However, in order to fulfil Eq. (5), the region $p_\mathrm {T}^{(V)}\ll M_V$ should be excluded from the reweighting procedure, unless QCD Sudakov logarithms are resummed to all orders in the theoretical calculations. Moreover, in order to simultaneously fulfill conditions (5) and (6), any aspect of the reconstructed vector-boson $p_\mathrm {T}$ that is better described at MC level should be excluded from the definition of *x* and included in ${\mathbf {y}}$. This applies, as discussed in Sect. 3, to multiple photon emissions off leptons, and to possible isolation prescriptions for the soft QCD radiation that surrounds leptons or photons. In general, purely non-perturbative aspects of MC simulations, i.e. MPI, UE, hadronization and hadron decays, should be systematically excluded from the definition of the reweighting variable *x*. Thus, impact and uncertainties related to this non-perturbative modeling will remain as in the original MC samples.

It should be stressed that the above considerations are meant for dark matter searches based on the *inclusive* MET distribution, while more exclusive searches that exploit additional information on hard jets may involve additional subtleties. In particular, for analyses that are sensitive to multi-jet emissions, using the inclusive vector-boson $p_\mathrm {T}$ as the reweighting variable would still fulfil Eq. (5), but the lack of QCD and EW corrections to $V+2$ jets production in MC simulations could lead to a violation of Eq. (6). In analyses that are sensitive to the tails of inclusive jet-$p_\mathrm {T}$ and $H_\mathrm {T}$ distributions this issue is very serious, and QCD+EW corrections should be directly implemented at MC level using multi-jet merging [19].

In general, as a sanity check of the reweighting procedure, we recommend verifying that, for reasonable choices of input parameters and QCD scales, (N)NLO QCD calculations and (N)LO merged MC predictions for vector-boson $p_\mathrm {T}$ distributions are in reasonably good agreement within the respective uncertainties. Otherwise, in case of significant MC mismodeling of the $\frac{\mathrm{d}}{\mathrm{d}x}\sigma ^{(V)}$ distribution, one should check the reliability of the MC in extrapolating TH predictions from the reweighting distribution to other relevant observables.

In general, one could check whether the one-dimensional reweighting via the variable *x* in Eq. (1) can in fact reproduce the dependence of the corrections in other kinematic variables that are relevant for the experimental analysis. To this end, distributions of $\sigma ^{(V)}$ w.r.t. another kinematic variable $x'$ should be calculated upon integrating Eq. (1). Switching on and off the corrections on the r.h.s. of Eq. (1) in $\sigma ^{(V)}_{\mathrm {TH}}$ and taking the ratio of the obtained differential cross sections $\sigma ^{(V)}$, produces the relative correction to the $x'$ distribution that could be compared to the corresponding result directly calculated from $\sigma ^{(V)}_{\mathrm {TH}}$.^3^

Finally, it is crucial to check that state-of-the-art predictions for absolute $\mathrm {d}\sigma /\mathrm {d}p_{\mathrm {T}}$ distributions agree with data for the various visible final states.

## Setup for numerical predictions

In this section we specify the physics objects (Sect. 3.1), acceptance cuts and observables (Sect. 3.2), input parameters (Sect. 3.3) and tools (Sect. 3.4) used in the theoretical calculations for $pp\rightarrow W^\pm /Z/\gamma +$ jet.

The definitions of physics objects, cuts and observables–which specify the setup for the reweighting procedure discussed in Sect. 2 – should be adopted both for theoretical calculations and for their Monte Carlo counterpart in the reweighting factor (3). The details of the reweighting setup are designed such as to avoid any possible deficit in the perturbative predictions (e.g. due to lack of resummation at small $p_\mathrm {T}$) and any bias due to non-perturbative aspects of Monte Carlo simulations (e.g. leptons and missing energy from hadron decays). Let us also recall that this setup is completely independent of the physics objects, cuts and observables employed in the experimental analyses.

As concerns input parameters and PDFs, the recommendation of Sect. 3.3 should be applied to all QCD and EW higher-order calculations. In particular, it is mandatory to compute (N)NLO QCD and EW corrections in the same EW input scheme, otherwise NLO EW accuracy would be spoiled. Instead, Monte Carlo simulations and the corresponding $\frac{\mathrm{d}}{\mathrm{d}x}\sigma ^{(V)}_{\mathrm {MC}}$ contributions to the reweighting factor (3) do not need to be based on the same input parameters and PDFs used for theory predictions.

We recommend handling $W/Z+\mathrm {jet}$ production and decay on the Monte Carlo side as the full processes $pp\rightarrow \ell \ell /\ell \nu /\nu \nu +\mathrm {jet}$, i.e. with a consistent treatment of off-shell effects, as is done on the theory side.

### Definition of physics objects

In the following we define the various physics objects relevant for higher-order perturbative calculations and for the reweighting in the Monte Carlo counterparts in Eq. (3).

#### Neutrinos

In parton-level calculations of $pp\rightarrow \ell \ell /\ell \nu /\nu \nu +\mathrm {jet}$, neutrinos originate only from vector-boson decays, while in Monte Carlo samples they can arise also from hadron decays. In order to avoid any bias in the reweighting procedure, only neutrinos arising from *Z* and *W* decays at Monte Carlo truth level should be considered.

#### Charged leptons

Distributions in the lepton $p_\mathrm {T}$ and other leptonic observables are known to be highly sensitive to QED radiative corrections, and the differences in the treatment of QED radiation on Monte Carlo and theory side can lead to a bias in the reweighting procedure. This should be avoided by using dressed leptons, i.e. recombining all leptons with nearly collinear photons that lie within a cone

7 $$\begin{aligned} \varDelta R_{\ell \gamma }=\sqrt{\varDelta \phi ^2_{\ell \gamma } +\varDelta \eta ^2_{\ell \gamma }}<R_{\mathrm {rec}}. \end{aligned}$$

For the radius of the recombination cone we employ the standard value $R_{\mathrm {rec}}=0.1$, which allows one to capture the bulk of the collinear final-state radiation, while keeping contamination from large-angle photon radiation at a negligible level. All lepton observables as well as the kinematics of reconstructed *W* and *Z* bosons are defined in terms of dressed leptons, and, in accordance with standard experimental practice, both muons and electrons should be dressed. In this way differences between electrons and muons, $\ell =e,\mu$, become negligible, and the reweighting function needs to be computed only once for a generic lepton flavor $\ell$.

Similarly as for neutrinos, only charged leptons that arise from *Z* and *W* decays at Monte Carlo truth level should be considered. Concerning QCD radiation in the vicinity of leptons, no lepton-isolation requirement should be imposed in the context of the reweighting procedure. Instead, in the experimental analysis lepton-isolation cuts can be applied in the usual manner.

#### *Z* and *W* bosons

The off-shell four-momenta of *W* and *Z* bosons are defined as

8 $$\begin{aligned} p^\mu _{W^+}= & {} p^\mu _{\ell ^+}+p^\mu _{\nu _\ell },\quad p^\mu _{W^-}=p^\mu _{\ell ^-}+p^\mu _{{\bar{\nu }}_\ell },\end{aligned}$$

9 $$\begin{aligned} p^\mu _{Z}= & {} p^\mu _{\ell ^+}+p^\mu _{\ell ^-},\quad p^\mu _{Z}=p^\mu _{\nu _\ell }+p^\mu _{{\bar{\nu }}_\ell }, \end{aligned}$$

where the leptons and neutrinos that result from *Z* and *W* decays are defined as discussed above.

#### Photons

At higher orders in QCD, photon production involves final-state $q\rightarrow q\gamma$ splittings that lead to collinear singularities when QCD radiation is emitted in the direction of the photon momentum. Since such singularities are of QED type, they are not canceled by corresponding virtual QCD singularities. Thus, in order to obtain finite predictions in perturbation theory, the definition of the $pp\rightarrow \gamma +$ jet cross section requires a photon-isolation prescription that vetoes collinear $q\rightarrow q\gamma$ radiation while preserving the cancellation of QCD infrared singularities.

To this end, in this study we adopt Frixione’s isolation prescription [27], which limits the hadronic transverse energy within a smooth cone around the photon by requiring

10 $$\begin{aligned} \sum \limits _{i=\mathrm{partons/hadrons}}&p_\mathrm{T, i}\,\varTheta (R-\varDelta R_{i\gamma })\nonumber \\&\le \epsilon _0\, p_\mathrm{T,\gamma } \left( \frac{1-\cos R}{1-\cos R_0}\right) ^n \quad \forall \; R\le R_0, \end{aligned}$$

where the sum runs over all quarks/gluons and hadrons at parton level and Monte Carlo level, respectively, while $p_\mathrm{T,i}$ and $p_\mathrm{T,\gamma }$ denote the transverse momenta of partons/hadrons and photons. The $p_\mathrm {T}$-fraction $\varepsilon _0$, the cone size $R_0$, and the exponent *n* are free parameters that allow one to control the amount of allowed QCD radiation in the vicinity of the photon.

The photon-isolation prescription is applicable to QCD as well as to EW higher-order corrections. At NLO EW, $\gamma +$ jet production involves bremsstrahlung contributions with two final-state photons. In this case, at least one isolated photon is required. The other photon might become soft, guaranteeing the cancellation of related soft and collinear singularities in the virtual EW corrections. In case of two isolated photons in the final state, the hardest photon is considered. In particular, an explicit photon-isolation prescription is mandatory at NLO EW in order to prevent uncanceled singularities from $q\rightarrow q\gamma$ splittings in the $\mathcal {O}(\alpha ^2\alpha _{\mathrm {S}})$ mixed EW–QCD contributions from $qq\rightarrow qq\gamma$ and crossing-related channels.

As a consequence of $q\rightarrow q\gamma$ collinear singularities and the need to apply a photon-isolation prescription, QCD corrections to $pp\rightarrow \gamma +$ jet behave differently as compared to $Z/W+$ jet production. Such differences can be important even at the TeV scale, where one might naively expect that massive and massless vector bosons behave in a universal way from the viewpoint of QCD dynamics. Instead, the presence of collinear $q\rightarrow q V$ singularities at (N)NLO QCD implies a logarithmic sensitivity to the vector-boson masses, which results, respectively, in $\ln (R_0)$ and $\ln (p_{\mathrm {T},V}/M_V)$ terms for the case of massless and massive vector bosons at $p_{\mathrm {T},V}\gg M_{W,Z}$.

A quantitative understanding of these differences and their implications on the correlation of QCD uncertainties between $\gamma +$ jet and $Z+$ jet production is crucial for the extrapolation of $\gamma +$ jet measurements to $Z+$ jet dark matter backgrounds. To this end, as discussed in Sect. 4, we propose a systematic approach based on the idea that, at large $p_{\mathrm {T},V}$, the $pp\rightarrow \gamma +$ jet process can be split into a dominant part with universal QCD dynamics (in the sense that QCD effects in $\gamma +$ jet and $Z/W+$ jet production are strongly correlated) and a remnant contribution that has to be handled as uncorrelated in the treatment of QCD uncertainties. To achieve this, we introduce a modified photon-isolation prescription, which is designed such as to render the QCD dynamics of $\gamma +$ jet and $Z/W+$ jet production as similar as possible at high $p_\mathrm {T}$. To this end we define a dynamic cone radius

11 $$\begin{aligned} R_{\mathrm {dyn}}(p_{\mathrm {T},\gamma },\varepsilon _0)=\frac{M_Z}{p_{\mathrm {T},\gamma }\sqrt{\varepsilon _0}}, \end{aligned}$$

which is chosen in such a way that the invariant mass of a photon-jet pair with $R_{\gamma j}=R_{\mathrm {dyn}}$ and $p_{\mathrm {T},j}=\varepsilon _0\, p_{\mathrm {T},\gamma }$ corresponds to the *Z*-boson mass, i.e.

12 $$\begin{aligned} M^2_{\gamma j}\simeq p_{\mathrm {T},\gamma }\,p_{\mathrm {T},j} R^2_{\gamma j}=\varepsilon _0\, p^2_{\mathrm {T},\gamma } R_{\mathrm {dyn}}^2 = M_Z^2, \end{aligned}$$

where the first identity is valid in the small-*R* approximation. In this way, using a smooth isolation with $R_0=R_{\mathrm {dyn}}(p_{\mathrm {T},\gamma },\varepsilon _0)$ mimics the role of the *Z*- and *W*-boson masses as regulators of collinear singularities in $Z/W+$jet production at high $p_\mathrm {T}$, while using a fixed cone radius $R_0$ would correspond to an effective $M_{\gamma j}$ cut well beyond $M_{Z,W}$, resulting in a more pronounced suppression of QCD radiation in $\gamma +$ jet production as compared to $Z/W+$ jet.

**Fig. 2:**
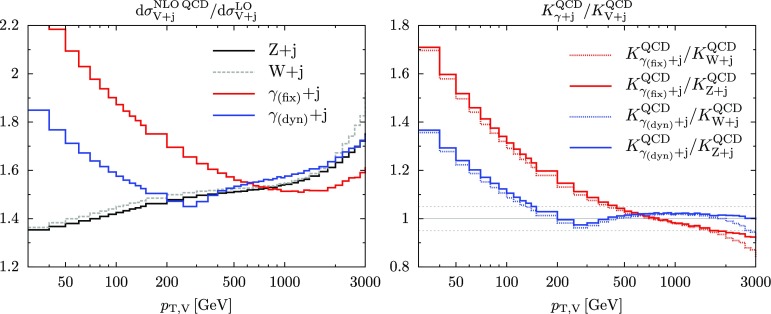
Comparison of NLO QCD *K*-factors (left) for $W+$ jet, $Z+$ jet, and $\gamma +$ jet production with dynamic photon isolation (13) and standard fixed-cone isolation (14). On the right corresponding ratios of *K*-factors are shown, and the dotted lines indicate *K*-factor variations of $\pm 0.05$

Specifically, as default photon selection for the theoretical predictions^4^ in this study we use the dynamic cone isolation defined through Eqs. (10) and (11), with parameters

13 $$\begin{aligned} \varepsilon _{0,\mathrm {dyn}}= & {} 0.1,\qquad n_\mathrm {dyn}=1, \nonumber \\ R_{0,\mathrm {dyn}}= & {} \mathrm {min}\left\{ 1.0, R_{\mathrm {dyn}}(p_{\mathrm {T},\gamma },\varepsilon _{\mathrm {dyn},0})\right\} . \end{aligned}$$

Note that, in order to prevent that the veto against collinear QCD radiation is applied to an excessively large region of phase space, the dynamic cone radius in Eq. (13) is limited to $R_{\mathrm {dyn}}\le 1.0$. As a result of this upper bound, for $p_{\mathrm {T},\gamma }< M_Z\varepsilon ^{-1/2}_{0,\mathrm {dyn}}\simeq 290$ GeV the cone radius is kept fixed, and the impact of collinear QCD radiation starts to be significantly enhanced as compared to the case of $Z/W+$ jet production. Vice versa, for $p_{\mathrm {T},\gamma }> M_Z\varepsilon ^{-1/2}_{0,\mathrm {dyn}}$, thanks to the dynamic isolation cone (13), QCD effects in $\gamma +$ jet and $Z/W+$ jet production become closely related, and the degree of correlation between QCD uncertainties across all $V+$ jet processes can be described with the prescription of Eqs. (37)–(38).

For a realistic assessment of theoretical uncertainties, one should also consider the fact that photon-isolation prescriptions used in experimental analyses differ in a significant way from the dynamic prescription of Eq. (13). To this end, we recommend to repeat the reweighting procedure using theory predictions for $\gamma +$ jet based on a standard Frixione isolation (10) with fixed cone radius and parameters that mimic typical experimental selections at particle level [28],

14 $$\begin{aligned} {\varepsilon }_{{0,\mathrm {fix}}}=0.025,\quad n_{\mathrm {fix}}=2,\quad R_{0,\mathrm {fix}}= 0.4. \end{aligned}$$

The difference between $\gamma +$ jet MC samples reweighted in the dynamic- and fixed-cone setup should be taken as an additional uncertainty for $pp\rightarrow \gamma +$ jet. As ingredients for this uncertainty estimate we provide higher-order QCD predictions (without uncertainties) with fixed-cone isolation (14) besides the full set of $pp\rightarrow \gamma +$ jet predictions and uncertainties with dynamic photon isolation (see Appendix A). In the EW corrections, differences between the two photon-isolation prescriptions are well below the percent level. Thus predictions for $\gamma +$ jet at (n)NLO EW are provided only with the dynamic cone prescription of Eq. (13).

In Fig. 2 we present a comparison of the NLO QCD *K*-factors for $W/Z+$ jet and $\gamma +$ jet production with dynamic and fixed-cone isolation. For $p_{\mathrm {T},\gamma }<290$ GeV, where both isolation prescriptions correspond to a fixed cone radius, the QCD corrections to $pp\rightarrow \gamma +$ jet grow rapidly with decreasing $p_{\mathrm {T}}$. At low $p_{\mathrm {T}}$, due to the smaller cone size, fixed isolation ($R_0=0.4$) leads to more pronounced corrections as compared to dynamic isolation ($R_0=1.0$), but the slopes of the corresponding $\gamma +$ jet *K*-factors are quite similar to each other and very different as compared to the ones for $pp\rightarrow W/Z+$ jet. In the case of fixed isolation, this difference persists also in the high-$p_{\mathrm {T}}$ regime (apart form the accidental agreement of *K*-factors at $p_{\mathrm {T},V}\approx 800$ GeV). Instead, in the case of dynamic photon isolation, at large $p_{\mathrm {T}}$ the QCD corrections to $\gamma +$ jet and $W/Z+$ jet production turn out to be remarkably similar, both in shape and size. As expected, the onset of this universal behavior is located close to $p_{\mathrm {T},\gamma }=290$ GeV, where the isolation radius $R_{0,\mathrm {dyn}}$ starts varying with $p_{\mathrm {T}}$ in a way that rejects QCD radiation with $M_{\gamma j}\lesssim M_{W,Z}$. The differences between $\gamma +$ jet and $W/Z+$ jet *K*-factors remain as small as a few percent up to the TeV scale.

#### QCD partons and photons inside jets

In order to avoid any bias due to the different modeling of jets in MC simulations and perturbative calculations, theory calculations and reweighting should be performed at the level of inclusive vector-boson $p_\mathrm {T}$ distributions, without imposing any requirement on the recoiling jet(s). Predictions presented in this study are thus independent of specific jet definitions or jet cuts.

**Fig. 3:**
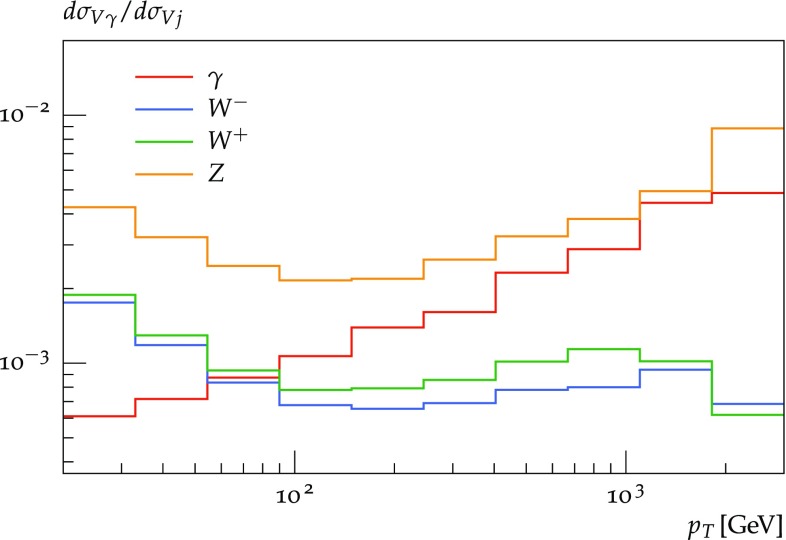
Ratios of distributions in the vector-boson transverse momenta for $pp\rightarrow V\gamma$ versus $pp\rightarrow Vj$ at LO with $\mu _{\mathrm {R},\mathrm {F}}=H_\mathrm {T}/2$. The vector bosons $V=W^\pm ,Z,\gamma$ are on shell and $\sqrt{s}=13\,\text {TeV}$

Concerning the composition of the recoil, we observe that, at NLO EW, $q\rightarrow q\gamma$ splittings can transfer an arbitrary fraction of the recoiling momentum from QCD partons to photons. In particular, in $pp\rightarrow V\gamma j$ contributions of $\mathcal {O}(\alpha ^2\alpha _{\mathrm {S}})$, the photon can carry up to 100% of the recoil momentum. Such contributions involve soft QCD singularities that are canceled by including also virtual QCD corrections to $pp\rightarrow V\gamma$. In order to minimize double counting with diboson production,^5^ $V\gamma$ production at LO is not included in the EW corrections to $pp\rightarrow Vj$. In practice, as demonstrated in Fig. 3, the relative weight of $pp\rightarrow V\gamma$ at $\mathcal {O}(\alpha ^2)$ versus $pp\rightarrow Vj$ at $\mathcal {O}(\alpha \alpha _{\mathrm {S}})$ is well below the percent level. Thus the impact of $\mathcal {O}(\alpha ^2\alpha _{\mathrm {S}})$ contributions from hard $V\gamma$ production, which are included in this study, should be completely negligible.

### Cuts and observables

Theoretical calculations and the reweighting of MC samples should be performed in a fully inclusive $V+$ jet setup, imposing a single cut

15 $$\begin{aligned} p_{\mathrm {T},V}>30\,\text {GeV} \quad \text{ for }\quad V=W^\pm ,Z,\gamma , \end{aligned}$$

with $p_{\mathrm {T},W^\pm }$ and $p_{\mathrm {T},Z}$ defined as in Sect. 3.1. The cut (15) is crucial in order to avoid the region where perturbative predictions suffer form the lack of QCD resummation.^6^

**Table 1:** Extra selection cuts, in addition to Eq. (15), and observables for the various $V+$ jet processes. Alternative predictions for $\gamma +$jet production are provided also for the case of a standard Frixione isolation with parameters (14)

Process	Extra cuts	Observable	Comments
$pp\rightarrow \ell ^+\nu _\ell +$ jet	None	$p_{\mathrm {T},\ell ^+\nu _\ell }$	$\ell = e$ or $\mu$
$pp\rightarrow \ell ^-{\bar{\nu }}_\ell +$ jet	None	$p_{\mathrm {T},\ell ^-{\bar{\nu }}_\ell }$	$\ell = e$ or $\mu$
$pp\rightarrow \nu _\ell {\bar{\nu }}_\ell +$ jet	None	$p_{\mathrm {T},\nu _\ell {\bar{\nu }}_\ell }$	$\ell =e+\mu +\tau$
$pp\rightarrow \ell ^+\ell ^-+$ jet	$m_{\ell \ell } > 30\,\text {GeV}$	$p_{\mathrm {T},\ell ^+\ell ^-}$	$\ell = e$ or $\mu$
$pp\rightarrow \gamma +$ jet	Dynamic isolation (11)–(13)	$p_{\mathrm {T},\gamma }$	

For leptons and MET we do not apply any $p_\mathrm {T}$ or rapidity cuts. Moreover, we do not impose any restrictions on QCD radiation in the vicinity of leptons and MET. Also QCD radiation is handled in a fully inclusive way, i.e. the presence of a recoiling jet is not explicitly required, and, as discussed in Sect. 3.1, at NLO EW the recoil can be entirely carried by a photon. Here we want to stress again that of course the particle-level analysis of the reweighted Monte Carlo samples can (and will) involve a more exclusive event selection than used for the reweighting itself.

**Fig. 4:**
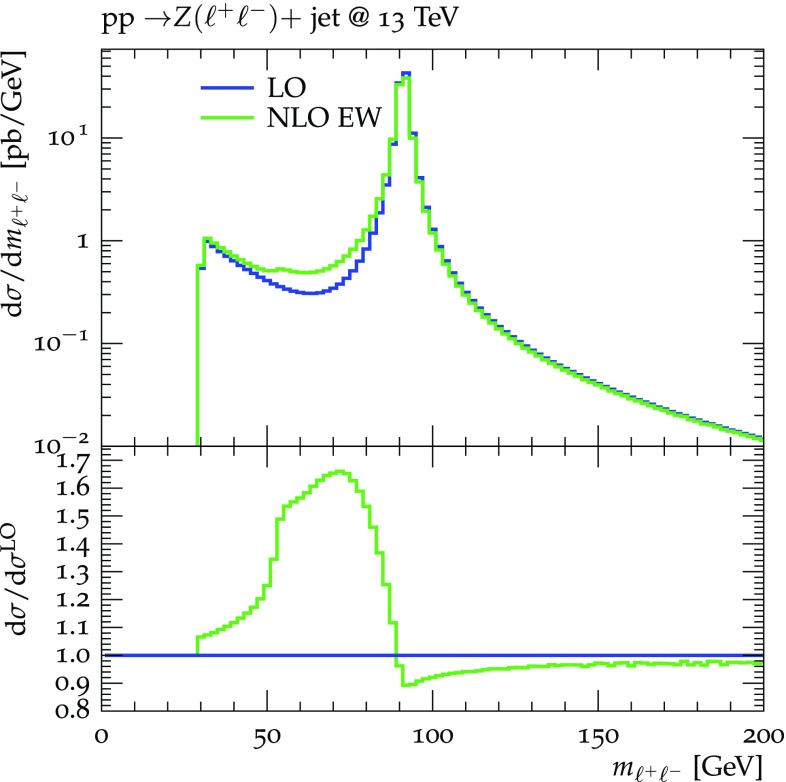
Dilepton invariant-mass distribution in $pp\rightarrow \ell ^+\ell ^-+$jet for $m_{\ell \ell }\in [30,200]\,\text {GeV}$ comparing LO and NLO EW. Collinear lepton–photon pairs with $R_{\gamma \ell } < 0.1$ are recombined

The differential distributions to be used for the reweighting of the various $pp\rightarrow V+$ jet processes and process-specific selection cuts to be applied in addition to Eq. (15) are summarized in Table 1. In the case of $pp\rightarrow \nu {\bar{\nu }}+$ jet all three neutrino species are added, while for all other *Z* and *W* decays only a single lepton generation is considered. For $pp\rightarrow \ell ^+\ell ^-+$ jet an extra invariant-mass cut is applied in order to avoid far off-shell contributions, especially from $\gamma ^*\rightarrow \ell ^+\ell ^-$ at low invariant mass. The relatively low value of the lower cut, $m_{\ell \ell }> 30\,\text {GeV}$, is intended to minimize cross section loss due to photon radiation that shifts events from the *Z*-peak region down to lower invariant mass (see Fig. 4). This choice guarantees a reduced sensitivity with respect to the modeling of QED radiation.

The following binning is adopted for distributions in the reconstructed vector-boson transverse momenta:

16 $$\begin{aligned}&\frac{p_{\mathrm {T}}}{\text {GeV}} \in \left[ 30, 40, \ldots , 140, 150, 200, 250 \ldots , 950, 1000,\right. \nonumber \\&\quad \left. 1100, 1200, 1300, 1400, 1600\ldots , 2800, 3000, 6500\right] .\nonumber \\ \end{aligned}$$

### Input parameters, PDFs and QCD scales

Input parameters and PDFs employed for theoretical predictions in this study are specified in the following. Let us recall that, as discussed in Sect. 2, Monte Carlo samples used in the experimental analyses do not need to be generated with the same input parameters and PDFs used for higher-order theoretical predictions.

In the calculation of $pp\rightarrow \ell \ell /\ell \nu /\nu \nu /\gamma \,+$ jet we use the gauge-boson masses [30]

17 $$\begin{aligned} M_Z= & {} 91.1876~\text {GeV},\quad M_W=80.385~\text {GeV}, \end{aligned}$$

and the corresponding widths,

18 $$\begin{aligned} \Gamma _Z= & {} 2.4955~\text {GeV},\quad \Gamma _W=2.0897~\text {GeV}. \end{aligned}$$

The latter are obtained from state-of-the-art theoretical calculations. For the top-quark [30] and Higgs-boson [31] masses and widths we use

19 $$\begin{aligned} M_t= & {} 173.2~\text {GeV},\quad M_H=125~\text {GeV}, \end{aligned}$$

and^7^

20 $$\begin{aligned} \Gamma _t= & {} 1.339~\text {GeV},\quad \Gamma _H=0~\text {GeV}. \end{aligned}$$

All unstable particles are treated in the complex-mass scheme [32], where width effects are absorbed into the complex-valued renormalized masses

21 $$\begin{aligned} \mu ^2_i=M_i^2-\mathrm {i}\Gamma _iM_i \quad \text{ for }\;i=W,Z,t. \end{aligned}$$

For $W+$jet and $Z+$jet production processes the EW couplings are derived from the gauge-boson masses and the Fermi constant, ${G_\mu }=1.16637\times 10^{-5}~\text {GeV} ^{-2}$, using

22 $$\begin{aligned} \alpha =\left| \frac{\sqrt{2}\sin ^2\theta _{\mathrm {w}}\,\mu ^2_W G_\mu }{\pi }\right| , \end{aligned}$$

while for $\gamma +$jet production the EW coupling is chosen to be [30]

23 $$\begin{aligned} \alpha =\alpha (0)=1/137.035999074. \end{aligned}$$

In both schemes the weak mixing angle $\theta _{\mathrm {w}}$ is determined by

24 $$\begin{aligned} \sin ^2\theta _{\mathrm {w}}=1-\cos ^2\theta _{\mathrm {w}}=1-\frac{\mu _W^2}{\mu _Z^2}, \end{aligned}$$

and it becomes complex-valued. The $G_\mu$-scheme guarantees an optimal description of pure SU(2) interactions at the EW scale. It is the scheme of choice for $W+$ jet production, and it provides a very good description of $Z\,+$ jet production as well. The $\alpha (0)$ scheme to be used for $\gamma +$jet, on the other hand, expresses the fact that on-shell photons effectively couple at a scale $Q^2=0$. The CKM matrix is assumed to be diagonal and we checked at LO and NLO QCD that for $W+$jet production the difference with respect to a non-diagonal CKM matrix is always well below 1%. For the choice of renormalization and factorization scales and variations thereof we refer to Sect. 4.1.

For the calculation of hadron-level cross sections at (N)NLO QCD + (n)NLO EW we employ the LUXqed_plus_PDF4LHC15_nnlo_100 PDF set, which is based on PDF4LHC NNLO PDFs [33–38] supplemented with QED effects [39]. The same PDF set, and the related $\alpha _{\mathrm {S}}$ value, is used throughout, i.e. also in the relevant LO and NLO ingredients used in the estimate of theoretical uncertainties. At the level of precision discussed in this study also the uncertainty on the value of $\alpha _{\mathrm {S}}$ becomes relevant. Given 1% uncertainty on the measured value of $\alpha _{\mathrm {S}}$ this results in an overall 1–2% normalization uncertainty on the differential $p_{\mathrm {T}}$ distributions. However, one should keep in mind that in the process ratios this uncertainty cancels completely and thus it is irrelevant for background estimates in DM searches at high-MET. Consistently with the five-flavor number scheme employed in the PDFs, *b*-quarks are treated as massless partons, and channels with initial-state *b*-quarks are taken into account. All light quarks, including bottom quarks, are treated as massless particles, and top-quark loops are included up to NLO throughout. Matrix elements at (N)NLO are evaluated using the five-flavor running of the strong coupling supported by the PDFs and, for consistency, top-quark loops are renormalized in the decoupling scheme. For the NNLO QCD coefficient no top-quark loops are considered.

For the assessment of PDF uncertainties the PDF4LHC prescription [33] is adopted. In addition to standard PDF variations, also additional LUXqed variations for the photon PDF are applied. For more details see more details in Sects. 4.3–4.4.

### Computational frameworks

The theoretical predictions presented in Sect. 4 include corrections up to NNLO QCD and NLO EW, as well as Sudakov EW effects at $\mathcal {O}(\alpha ^2)$. They have been obtained by means of a variety of methods and tools, as detailed in the following.

The NLO QCD and NLO EW calculations for all $pp\rightarrow V+$ jet processes have been performed with Munich+OpenLoops and/or Sherpa+OpenLoops. In these automated frameworks [19, 40, 41] virtual amplitudes are provided by the OpenLoops program [42, 43], combined with the Collier tensor reduction library [44] or with CutTools [45]. The remaining tasks are supported by the two independent and fully automated Monte Carlo generators Munich  [46] and Sherpa  [47–50]. Additionally, we carefully validated the NLO EW predictions against the results of Refs. [17, 18, 20]. The NLO EW calculations for $pp\rightarrow V+2$ jets performed to test the factorization of QCD and EW corrections have been checked against the one of Ref. [51] for $pp\rightarrow Z+2$ jets in Ref. [21]. The NLO EW amplitudes for all $V+$jet processes in OpenLoops have been supplemented with the one- and two-loop analytical Sudakov logarithms of Refs. [22–25, 52].

The NNLO QCD predictions for $Z+$jet production have been obtained with the parton-level event generator NNLOjet, which provides the necessary infrastructure to perform fully differential calculations at NNLO using the antenna subtraction formalism [53–61]. The computation of $pp\rightarrow W+$jet through NNLO is based on the *N*-jettiness subtraction scheme for NNLO calculations [13]. The above-cut contribution within the *N*-jettiness subtraction was obtained using Munich+OpenLoops. The NNLO QCD prediction for the $pp\rightarrow \gamma +$jet process is based on the calculations of Refs. [15, 16] and has been obtained using MCFM [62]. In order to ensure the correctness of the numerical implementation of cuts and other parameters in the NNLO codes, a detailed comparison has been performed at the level of the NLO QCD results as described above.

## Higher-order QCD and EW predictions

Precise theory predictions for $V+$ jet production require QCD and EW higher-order corrections, mixed QCD–EW contributions, as well as photon-induced contributions,

25 $$\begin{aligned} \frac{\mathrm{d}}{\mathrm{d}x}\sigma ^{(V)}_{\mathrm {TH}}= \frac{\mathrm{d}}{\mathrm{d}x}\sigma ^{(V)}_{{\mathrm {QCD}}}+ \frac{\mathrm{d}}{\mathrm{d}x}\varDelta \sigma ^{(V)}_{{\mathrm {EW}}} + \frac{\mathrm{d}}{\mathrm{d}x}\varDelta \sigma ^{(V)}_{{\mathrm {mix}}} + \frac{\mathrm{d}}{\mathrm{d}x}\sigma ^{(V)}_{\gamma -\mathrm{ind.}}. \end{aligned}$$

In this section we present theoretical predictions that include corrections up to NNLO QCD and NLO EW supplemented by EW Sudakov logarithms at two loops. Moreover, we introduce a coherent theoretical framework for the combination of EW and QCD calculations for the various $V+$ jet production processes and for the assessment of the corresponding remaining sources of theoretical uncertainty. State-of-the-art QCD and EW predictions and the related theoretical uncertainties are discussed in Sects. 4.1 and 4.2, respectively. Section 4.3 is devoted to photon-induced channels and Sect. 4.4 to PDF uncertainties, while in Sect. 4.5 we discuss the real emission of vector bosons, and mixed corrections of $\mathcal {O}(\alpha \alpha _{\mathrm {S}})$ are addressed in Sect. 4.6 by means of a factorized combination of QCD and EW corrections.

To illustrate the effect of higher-order corrections and uncertainties we present a series of numerical results for $pp\rightarrow V+$ jet at a center-of-mass energy of 13 TeV in the setup specified in Section 3. In particular, $pp\rightarrow \gamma +$ jet predictions are based on the dynamic photon isolation (13). As anticipated in Sect. 3.1, this prescription provides a very convenient basis for the systematic modeling of the correlation of QCD uncertainties between the various $V+$ jet production processes (see Sect. 4.1).

Vector-boson $p_\mathrm {T}$ spectra are plotted starting at 80 GeV, but for the sake of a complete documentation data sets are provided above 30 GeV (see Appendix A). However, we note that in the region of $p_\mathrm {T}\lesssim 100$ GeV there are potential sources of systematics that we are not controlling or even discussing, as they would require a separate study. These arise from the resummation of QCD Sudakov logarithms or from non-perturbative effects (e.g. an order $\Lambda _{\mathrm {QCD}}$ average shift of the vector-boson $p_\mathrm {T}$ associated with the asymmetry of color flow in the final state). Furthermore, as shown later, a reliable correlation between the *Z* / *W* spectra and the photon spectrum requires $p_\mathrm {T}$ to be large enough so that fragmentation contributions in $\gamma +$jet production become small. We also expect that in the $p_\mathrm {T}$ regions up to a few hundred GeV the statistics are sufficient to guarantee that experimental analyses of missing-$E_\mathrm {T}$ backgrounds can entirely rely on the direct measurement of the *Z* spectrum measured via $Z\rightarrow \ell ^+\ell ^-$. As a result, we believe that our conclusions on the systematic uncertainties are most reliable and useful for experimental applications in the region of $p_\mathrm {T}$ larger than 100–200 GeV.

### Higher-order QCD predictions

For perturbative QCD predictions at LO, NLO and NNLO we use the generic notation

26 $$\begin{aligned} \frac{\mathrm{d}}{\mathrm{d}x}\sigma ^{(V)}_{{\mathrm {QCD}}} = \frac{\mathrm{d}}{\mathrm{d}x}\sigma ^{(V)}_{\mathrm {N}^k\mathrm {LO}\,{\mathrm {QCD}}}, \end{aligned}$$

with $k=0,1$ or 2. Wherever possible, nominal predictions are provided at NNLO QCD, i.e. including terms up to^8^ $\mathcal {O}(\alpha \alpha _{\mathrm {S}}^3)$. However, as ingredients for the assessment of some theory uncertainties, also LO and NLO QCD contributions will be used.

For convenience, results at $\mathrm {N}^k\mathrm {LO}$ QCD are systematically expressed in terms of LO predictions and relative correction factors defined through

27 $$\begin{aligned} \frac{\mathrm{d}}{\mathrm{d}x}\sigma ^{(V)}_{\mathrm {N}^k\mathrm {LO}\,{\mathrm {QCD}}}({\varvec{\mu }})= & {} K^{(V)}_{\mathrm {N}^k\mathrm {LO}}(x,{\varvec{\mu }}) \frac{\mathrm{d}}{\mathrm{d}x}\sigma ^{(V)}_{\text {LO} \,{\mathrm {QCD}}}({\varvec{\mu }}_0). \end{aligned}$$

We calculate all $\mathrm {N}^k\mathrm {LO}$ and LO cross sections with one and the same set of NNLO PDFs as discussed in Sect. 3.3. The dependence on the renormalization and factorization scales, ${\varvec{\mu }}=(\mu _{R},\mu _{F})$, is absorbed into the *K*-factors, while LO predictions on the r.h.s. of Eq. (27) are taken at the central scale, ${\varvec{\mu }}_0=(\mu _{R,0},\mu _{F,0})$. For the central scale we adopt the commonly used choice

28 $$\begin{aligned} \mu _{R,0}=\mu _{F,0}=\mu _{0}=\hat{H}_{\mathrm {T}}'/2 , \end{aligned}$$

where the total transverse energy, $\hat{H}_{\mathrm {T}}'$, is defined as the scalar sum of the transverse energy of all parton-level final-state objects,

29 $$\begin{aligned} \hat{H}_{\mathrm {T}}'= E_{\mathrm {T},V}\,+\sum _{i\in \{\mathrm {q,g,\gamma }\}} |p_{\mathrm {T},i}|. \end{aligned}$$

Also quarks (q), gluons (g) and photons that are radiated in the (N)NLO QCD or EW corrections are included in $\hat{H}_{\mathrm {T}}'$, and the vector-boson transverse energy, $E_{\mathrm {T},V}$, is computed using the total (off-shell) four-momentum of the corresponding decay products, i.e.

30 $$\begin{aligned} E^2_{\mathrm {T},Z}= & {} p^2_{\mathrm {T},\ell ^+\ell ^-}+m_{\ell ^+\ell ^-}^2,\nonumber \\ E^2_{\mathrm {T},W}= & {} p^2_{\mathrm {T},\ell \nu }+m_{\ell \nu }^2,\nonumber \\ E^2_{\mathrm {T},\gamma }= & {} p^2_{\mathrm {T},\gamma }. \end{aligned}$$

In order to guarantee infrared safety at NLO EW, the scale (29) must be insensitive to collinear photon emissions off charged fermions. To this end, the vector-boson transverse energies defined in Eq. (30) should be computed in terms of dressed leptons as specified in Sect. 3.1, while $|p_{\mathrm {T},\gamma }|$ contributions to Eq. (29) should involve only photons that have not been recombined with charged leptons. It is worth to note that $\mu _{0} \approx p_{\mathrm {T},V}$ at large $p_{\mathrm {T},V}$.

#### Pure QCD uncertainties

The uncertainty associated with the truncation of the perturbative expansion in $\alpha _{\mathrm {S}}$ is estimated by means of factorization and renormalization scale variations. We consider standard seven-point variations applying, respectively, factor-2 rescalings, i.e.

31 $$\begin{aligned} \frac{{\varvec{\mu }}_i}{\mu _0}= & {} (1,1), (2,2), (0.5,0.5), (2,1), (1,2), (1,0.5), (0.5,1),\nonumber \\ \end{aligned}$$

where $i=0,\ldots 6$. Nominal predictions and related uncertainties are defined as the center and the half-width of the band resulting from the above variations. In terms of *K*-factors this corresponds to

32 $$\begin{aligned} K^{(V)}_{\mathrm {N}^k\mathrm {LO}}(x)= & {} \frac{1}{2}\left[ K^{(V,\mathrm {max})}_{\mathrm {N}^k\mathrm {LO}}(x)+K^{(V,\mathrm {min})}_{\mathrm {N}^k\mathrm {LO}}(x)\right] , \end{aligned}$$

33 $$\begin{aligned} \delta ^{(1)} K^{(V)}_{\mathrm {N}^k\mathrm {LO}}(x)= & {} \frac{1}{2}\left[ K^{(V,\mathrm {max})}_{\mathrm {N}^k\mathrm {LO}}(x)-K^{(V,\mathrm {min})}_{\mathrm {N}^k\mathrm {LO}}(x)\right] , \end{aligned}$$

with

34 $$\begin{aligned} K_{\mathrm {N}^k\mathrm {LO}}^{(V,\mathrm {max})}(x)= & {} \mathrm {max}\left\{ K^{(V)}_{\mathrm {N}^k\mathrm {LO}}(x,{\varvec{\mu }}_i)\left| 0\le i\le 6\right. \right\} ,\nonumber \\ K_{\mathrm {N}^k\mathrm {LO}}^{(V,\mathrm {min})}(x)= & {} \mathrm {min}\left\{ K^{(V)}_{\mathrm {N}^k\mathrm {LO}}(x,{\varvec{\mu }}_i)\left| 0\le i\le 6\right. \right\} . \end{aligned}$$

Since the shift resulting form the symmetrization of scale variations in Eq. (32) is encoded in the *K*-factors, also the LO *K*-factor differs from 1.

**Fig. 5:**
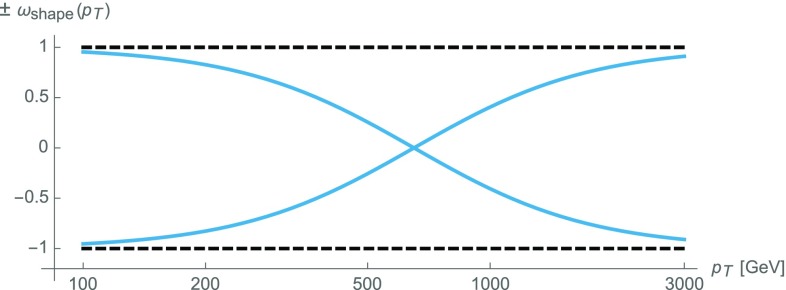
Shape variation function $\omega _\mathrm {shape}(p_\mathrm {T})$ defined in Eq. (36)

Constant scale variations mainly affect the overall normalization of $p_\mathrm {T}$-distributions and tend to underestimate shape uncertainties, which play an important role in the extrapolation of low-$p_\mathrm {T}$ measurements to high $p_\mathrm {T}$. Thus, for a reasonably conservative estimate of shape uncertainties, we introduce an additional variation,

35 $$\begin{aligned} \delta ^{(2)} K^{(V)}_{\mathrm {N}^k\mathrm {LO}}(x)= \omega _\mathrm {shape}(x)\, \delta ^{(1)} K^{(V)}_{\mathrm {N}^k\mathrm {LO}}(x), \end{aligned}$$

where the standard scale uncertainty (33) is supplemented by a shape distortion $\omega _\mathrm {shape}(x)$, with $|\omega _\mathrm {shape}(x)|\le 1$ and $\omega _\mathrm {shape}(x)\rightarrow \pm 1$ at high and small transverse momentum, respectively. The function $\omega _\mathrm {shape}$ is defined as

36 $$\begin{aligned} \omega _\mathrm {shape}(p_\mathrm {T})= \tanh \left[ \ln \left( \frac{p_\mathrm {T}}{p_{\mathrm {T},0}}\right) \right] =\frac{p^2_\mathrm {T}-p^2_{\mathrm {T},0}}{p^2_\mathrm {T}+p^2_{\mathrm {T},0}}, \end{aligned}$$

and as reference transverse momentum we choose the value $p_{\mathrm {T},0}=650$ GeV, which corresponds (in logarithmic scale) to the middle of the range of interest, 0.2–2 TeV. As illustrated in Fig. 5, the function $\omega _\mathrm {shape}(x)$ induces asymmetric variations that cover $\pm 75\%$ of the standard scale variation band for $p_\mathrm {T}\in [250,1750]\,\text {GeV}$. Note that, in the combination of the uncertainties (33) and (35), our choice to have an additional shape variation augments the standard scale uncertainty by a factor $1 \le \sqrt{1+\omega _\mathrm {shape}^2(p_\mathrm {T})}\le \sqrt{2}$.

Besides shape uncertainties, also the correlation of QCD uncertainties across $V+$ jet processes plays a key role in fits of the $Z(\nu {\bar{\nu }})+$ jet dark matter background, and the quantitative understanding of such process correlations belongs to the most important theoretical aspects in dark matter searches. From the viewpoint of QCD interactions, the processes $pp\rightarrow W+$ jet and $pp\rightarrow Z+$ jet are quite similar to each other at $p_{\mathrm {T},V}\gg M_{W,Z}$. Thus, the respective QCD uncertainties are expected to be strongly correlated. However, due to the presence of $q\rightarrow q\gamma$ collinear singularities and the need to suppress them with an appropriate photon-isolation prescription, higher-order QCD contributions to $\gamma +$ jet production can behave in a significantly different way as compared to the case of $pp\rightarrow W/Z+$ jet. In order to reduce such differences, we adopt the dynamic photon-isolation approach defined in Eq. (13). As discussed in Sect. 3.1, this prescription renders the QCD dynamics of $pp\rightarrow \gamma +$ jet and $pp\rightarrow Z/W+$ jet processes almost universal. As a result, QCD *K*-factors $K^{(V)}_{\mathrm {N}^k\mathrm {LO}}(x)$ and their uncertainties $\delta ^{(i)}K^{(V)}_{\mathrm {N}^k\mathrm {LO}}(x)$ depend only very weakly^9^ on *V* at high $p_\mathrm {T}$, and in this situation the small process-dependent part of QCD *K*-factors can be used as an estimator of the degree of correlation across processes. To this end we consider the highest available term in the perturbative expansion,

37 $$\begin{aligned} \varDelta K^{(V)}_{\mathrm {N}^k\mathrm {LO}}(x)=K^{(V)}_{\mathrm {N}^k\mathrm {LO}}(x)/K^{(V)}_{\mathrm {N}^{k-1}\mathrm {LO}}(x)-1, \end{aligned}$$

and as estimate of unknown process-correlation effects we take the difference of the known QCD *K*-factors with respect to $Z+$ jet production,

38 $$\begin{aligned} \delta ^{(3)} K^{(V)}_{\mathrm {N}^k\mathrm {LO}}(x)= & {} \varDelta K^{(V)}_{\mathrm {N}^k\mathrm {LO}}(x)-\varDelta K^{(Z)}_{\mathrm {N}^k\mathrm {LO}}(x). \end{aligned}$$

This process-correlation uncertainty can be assessed using the central scale (28) throughout. Applying it to nominal predictions, i.e. replacing $K^{(V)}_{\mathrm {N}^k\mathrm {LO}} \rightarrow K^{(V)}_{\mathrm {N}^k\mathrm {LO}}\pm \delta ^{(3)} K^{(V)}_{\mathrm {N}^k\mathrm {LO}}$, amounts to doubling or removing *K*-factor differences between processes. The choice of $Z+$ jet production as reference process in Eq. (38) is arbitrary, but changing the reference process has very little impact on process correlations since the resulting overall shift in $\delta ^{(3)} K^{(V)}_{\mathrm {N}^k\mathrm {LO}}(x)$ cancels to a large extent in ratios of $V+$ jet cross sections.

The above prescription should be regarded as conservative, since parts of the available *K*-factors are downgraded from the status of known higher-order corrections to uncertainties. However, thanks to the fact that the $V+$ jet *K*-factors of the same order *k* are strongly correlated, $\delta ^{(3)} K^{(V)}_{\mathrm {N}^k\mathrm {LO}}(x) \ll \varDelta K^{(V)}_{\mathrm {N}^k\mathrm {LO}}$, the resulting losses of accuracy in the nominal $\mathrm {N}^k\mathrm {LO}$ predictions for individual processes are rather small.

For the application to experimental analyses, it is important to keep in mind that the above modeling of process correlations assumes a close similarity of QCD effects between all $pp\rightarrow V+$ jet processes, which is achieved, in the present study, by means of the dynamic photon isolation (13). Thus, as discussed in Sect. 3.1, experimental analyses that employ a different photon-isolation approach require an additional $\gamma +$ jet specific uncertainty.

The above uncertainties can be parametrized through a set of independent nuisance parameters, ${\varvec{\varepsilon }}_{\mathrm {QCD}}$, and combined using

39 $$\begin{aligned}&\frac{\mathrm{d}}{\mathrm{d}x}\sigma ^{(V)}_{\mathrm {N}^k\mathrm {LO}\,{\mathrm {QCD}}}({\varvec{\varepsilon }}_{{\mathrm {QCD}}})\nonumber \\&\quad = \left[ K^{(V)}_{\mathrm {N}^k\mathrm {LO}}(x)+\sum _{i=1}^3\varepsilon _{{\mathrm {QCD}},i}\,\delta ^{(i)} K^{(V)}_{\mathrm {N}^k\mathrm {LO}}(x) \right] \nonumber \\&\qquad \times \,\frac{\mathrm{d}}{\mathrm{d}x}\sigma ^{(V)}_{\text {LO} \,{\mathrm {QCD}}}({\varvec{\mu }}_0). \end{aligned}$$

The nuisance parameters $\varepsilon _{{\mathrm {QCD}},1},\varepsilon _{{\mathrm {QCD}},2}$ and $\varepsilon _{{\mathrm {QCD}},3}$ should be Gaussian distributed with one standard deviation corresponding to the range $\varepsilon _{{\mathrm {QCD}},i}\in [-1,+1]$. These parameters should be kept uncorrelated, but each $\varepsilon _{{\mathrm {QCD}},i}$-variation should be applied in a correlated way across $p_\mathrm {T}$ bins and processes, since correlation effects are consistently implemented in the $\delta ^{(i)} K^{(V)}_{\mathrm {N}^k\mathrm {LO}}(x)$ terms.

#### Numerical results

**Fig. 6:**
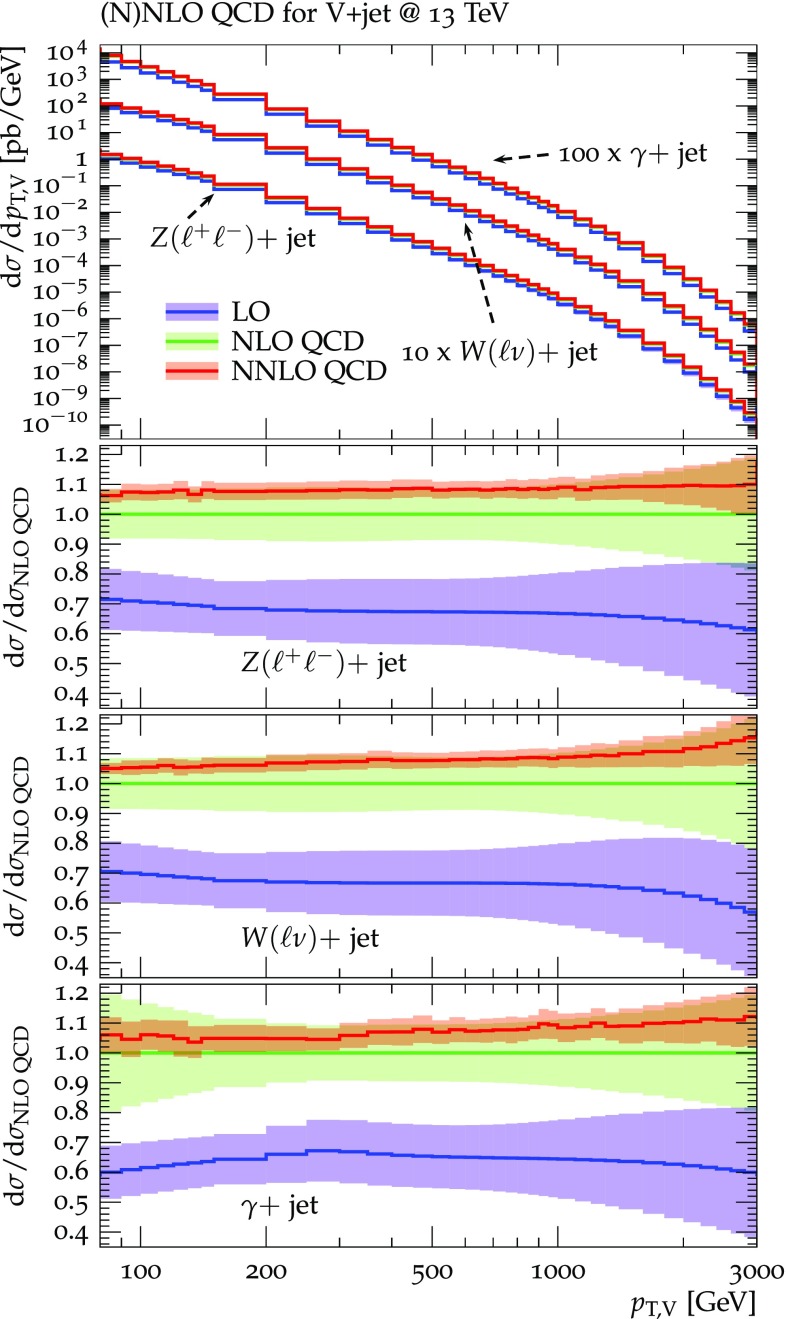
Higher-order QCD predictions and uncertainties for $Z(\ell ^+\ell ^-)+$jet, $W^\pm (\ell \nu )+$jet, and $\gamma +$jet production at 13 TeV. Absolute predictions at LO, NLO and NNLO QCD are displayed in the main frame. The ratio plots show results for individual processes normalized to NLO QCD. The bands correspond to the combination (in quadrature) of the three types of QCD uncertainties, $\delta ^{(i)}K_{\mathrm {N}^k\mathrm {LO}}$, i.e. scale uncertainties according to Eq. (33), shape uncertainties according to Eq. (35), and process-correlation uncertainties according to Eq. (38)

**Fig. 7:**
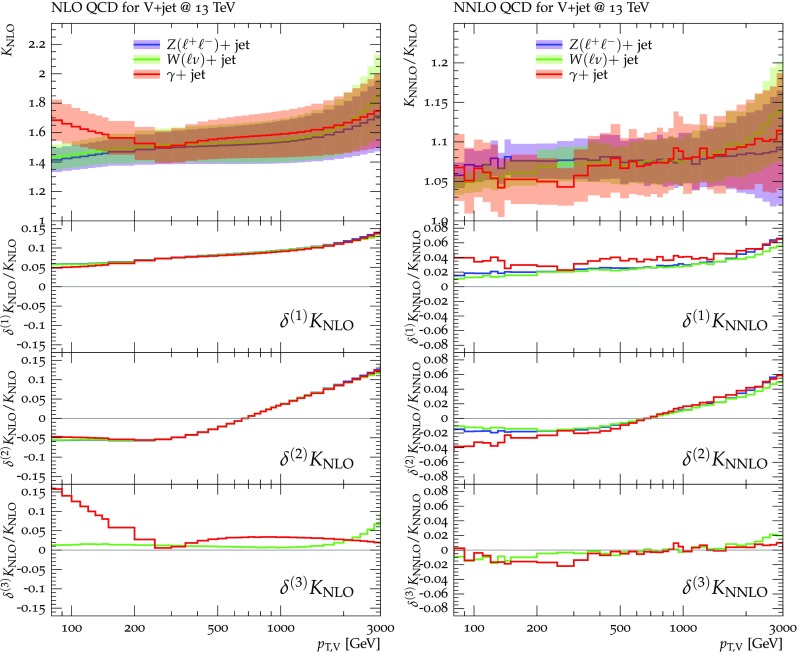
QCD *K*-factors at NLO (with respect to LO) on the left and at NNLO (with respect to NLO) on the right for the various $pp\rightarrow V+$ jet processes at 13 TeV. The bands in the upper frame correspond to scale variations, i.e. $\delta ^{(1)}K_\mathrm{NLO}$ and $\delta ^{(1)}K_{\mathrm {NNLO}}$. The lower frames show the individual uncertainties defined in Eqs. (33), (35), and (38). They are displayed as ratios $\delta ^{(i)}K_{\mathrm {N}^k\mathrm {LO}}/K_{\mathrm {N}^k\mathrm {LO}}$, which corresponds to the relative impact of uncertainties on $p_\mathrm {T}$ distributions at $\text {NLO}$ and $\mathrm {NNLO}$

**Fig. 8:**
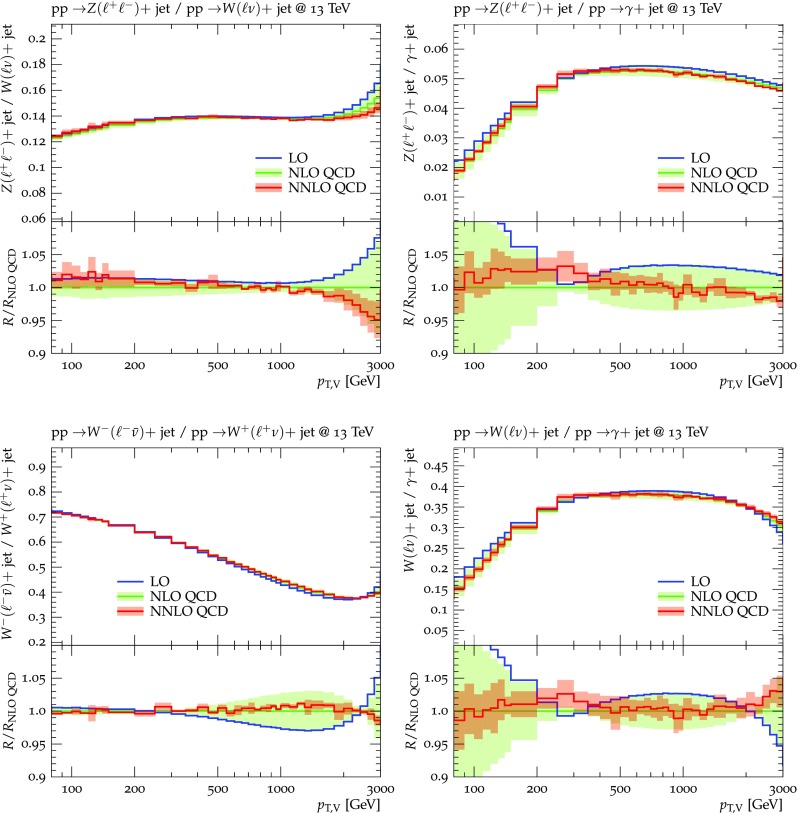
Ratios of $p_\mathrm {T}$-distributions for various $pp\rightarrow V+$jet processes at LO, NLO and NNLO QCD. The NLO and NNLO QCD uncertainties, estimated according to Eqs. (33), (35), and (38) are correlated amongst processes as described in the text and combined in quadrature. At LO only nominal predictions are shown

Predictions for $V+$jet distributions and their ratios at LO, NLO and NNLO QCD are presented in Figs. 6, 7 and 8 as well as in Figs. 18 and 19 (see Appendix B). In Figs. 7, 18 and 19, scale uncertainties (33), shape uncertainties (35), and process-correlation uncertainties (38) are shown separately, while in Figs. 6 and 8 the three QCD uncertainties are combined in quadrature. Here and in the following *W* denotes $W^+$ and $W^-$ combined.

At high transverse momentum, we find that QCD corrections and uncertainties for the various $V+\,$jet production processes behave in a very similar way. At NLO the corrections amount to 40–60% with residual uncertainties around 10–20%, while NNLO corrections increase the cross section by 5–10% and reduce the combined uncertainty to 3–10%. Scale variations $\delta ^{(1)}K_{\mathrm {N}^k\mathrm {LO}}$ and shape variations $\delta ^{(2)}K_{\mathrm {N}^k\mathrm {LO}}$ are the dominant sources of uncertainty in $p_{\mathrm {T}}$-distributions. Their contributions are very similar across $V+\,$jet processes. Thus in the ratios scale and shape variations largely cancel, and the process-correlation uncertainty $\delta ^{(3)}K_{\mathrm {N}^k\mathrm {LO}}$ tends to dominate.

The ratio plots (Fig. 8) allow one to appreciate small differences in the QCD dynamics of the various $V+$ jet processes. As reflected in the *Z* / *W* ratio, the NLO and NNLO corrections for the corresponding processes are almost identical, with differences below 1–2% up to one TeV. Only at very large $p_{\mathrm {T}}$ the NLO and also NNLO corrections to $W+$jet grow faster than in the case of $Z+$jet. This results in an increase of the process-correlation uncertainty $\delta ^{(3)}K_{\text {NLO}}$ up to about $5\%$ beyond $p_{\mathrm {T}} =2~\text {TeV}$.

As can be seen in the $Z/\gamma$ and $W/\gamma$ ratios, the higher-order QCD corrections to $\gamma +$jet production behave very similarly as for $Z+$ jet and $W+$ jet production at large $p_{\mathrm {T}}$. This is the result of the dynamic photon isolation (13), which guarantees that the differences in the NLO and NNLO corrections remain below 3–4% for $p_{\mathrm {T}} >200\,$ GeV. Instead, at lower $p_{\mathrm {T}}$ the behavior of $\gamma +$ jet production changes drastically due to mass effects, which results in sizable process-correlation uncertainties.^10^ Note that for $p_{\mathrm {T}} \approx 300~\text {GeV}$ the NLO process-correlation uncertainty in $pp\rightarrow \gamma +$jet is accidentally very small (see Fig. 18) yielding a pinch in the total QCD uncertainty for the $Z/\gamma$ and the $W/\gamma$ ratios (see also Fig. 19). However, one should keep in mind that an additional analysis-dependent photon-isolation uncertainty (see Sect. 3.1) has to be considered for these ratios.

In general, comparing QCD predictions at different orders we observe a good convergence of the perturbative expansion, and the fact that process ratios receive very small corrections both at NLO and NNLO provides strong evidence for the universality of QCD dynamics is all $V+$ jet processes. Results at NNLO provide also a crucial test of the goodness of the proposed approach for the estimate of QCD uncertainties and their correlations. In particular, the remarkable consistency between NNLO and NLO predictions in Fig. 8 confirms that QCD uncertainties for process ratios are as small as 1–2%.

### Electroweak corrections

For EW higher-order corrections we use the notation

40 $$\begin{aligned} \frac{\mathrm{d}}{\mathrm{d}x}\sigma ^{(V)}_{\text {NLO} \,{\mathrm {EW}}}= & {} \frac{\mathrm{d}}{\mathrm{d}x}\sigma ^{(V)}_{\text {LO} \,{\mathrm {QCD}}}+ \frac{\mathrm{d}}{\mathrm{d}x}\varDelta \sigma ^{(V)}_{\text {NLO} \,{\mathrm {EW}}}, \nonumber \\ \frac{\mathrm{d}}{\mathrm{d}x}\sigma ^{(V)}_{\mathrm {nNLO}\,{\mathrm {EW}}}= & {} \frac{\mathrm{d}}{\mathrm{d}x}\sigma ^{(V)}_{\text {NLO} \,{\mathrm {EW}}}+ \frac{\mathrm{d}}{\mathrm{d}x}\varDelta \sigma ^{(V)}_{{\mathrm {NNLO}}\,\mathrm {Sud}}, \end{aligned}$$

where $\varDelta \sigma ^{(V)}_{\text {NLO} \,{\mathrm {EW}}}$ denotes exact $\mathcal {O}(\alpha ^2\alpha _{\mathrm {S}})$ contributions, and ‘NNLO Sud’ stands for $\mathcal {O}(\alpha ^3\alpha _{\mathrm {S}})$ EW Sudakov logarithms in NLL approximation (see below). Their combination is dubbed nNLO EW as it accounts for the dominant EW effects at NNLO. While our power counting does not consider the extra factor $\alpha$ associated with vector-boson decays, all predictions for $pp\rightarrow W/Z+$ jet at (N)NLO QCD + NLO EW are at the level of the full processes, $pp\rightarrow \ell \nu /\ell \ell /\nu \nu +$ jet, including off-shell effects and NLO EW corrections in decays. Since EW Sudakov logarithms do not enter *W* and *Z* decays, they are applied only at the level of $pp\rightarrow V+$ jet production, including off-shell decays at LO.

The EW corrections, similarly as for the QCD ones, are also expressed in terms of correction factors with respect to LO QCD,

41 $$\begin{aligned} \frac{\mathrm{d}}{\mathrm{d}x}\sigma ^{(V)}_{{\mathrm {EW}}}({\varvec{\mu }})= & {} \left[ 1+\kappa ^{(V)}_{{\mathrm {EW}}}(x,{\varvec{\mu }})\right] \frac{\mathrm{d}}{\mathrm{d}x}\sigma ^{(V)}_{\text {LO} \,{\mathrm {QCD}}}({\varvec{\mu }}), \end{aligned}$$

where EW stands for NLO EW or nNLO EW. At variance with Eq. (27), here the EW $\kappa$-factors are defined by taking the factorized LO cross section at the same QCD scales, ${\varvec{\mu }}=(\mu _{\mathrm {R}},\mu _{\mathrm {F}})$, as in the higher-order EW prediction. In this way, since QCD scale variations at LO QCD and (n)NLO EW have almost identical impact, the relative EW correction is essentially independent of ${\varvec{\mu }}$. Thus, in practice, $\kappa _{\mathrm {EW}}$ can be computed at the fixed reference scale,

42 $$\begin{aligned} \kappa ^{(V)}_{\mathrm {EW}}(x,{\varvec{\mu }})\simeq \kappa ^{(V)}_{\mathrm {EW}}(x,{\varvec{\mu }}_0)= \kappa ^{(V)}_{\mathrm {EW}}(x), \end{aligned}$$

while the scale dependence of $\sigma ^{(V)}_{{\mathrm {EW}}}$ is generated through $\sigma ^{(V)}_{\text {LO} \,{\mathrm {QCD}}}({\varvec{\mu }})$ in Eq. (41). Moreover, the EW correction factor $\kappa ^{(V)}_{\mathrm {EW}}$ is rather insensitive to the choice of PDF set as long as it is derived from cross sections that are based on the same PDFs. Analogously to Eq. (40), nNLO EW correction factors are split into a full NLO part and an NNLO Sudakov part,

43 $$\begin{aligned} \kappa ^{(V)}_{\mathrm {nNLO}\,{\mathrm {EW}}}(x)= \kappa ^{(V)}_{\text {NLO} \,{\mathrm {EW}}}(x)+\kappa ^{(V)}_{\mathrm {NNLO}\,\mathrm {Sud}}(x). \end{aligned}$$

At NLO EW, all relevant contributions of $\mathcal {O}{(\alpha ^2\alpha _{\mathrm {S}})}$ are included. In the $q{\bar{q}}$ channel, and in all crossing-related channels, they comprise the following types of corrections:

- virtual EW corrections to $q{\bar{q}}\rightarrow Vg$;
- $$q{\bar{q}}\rightarrow Vg\gamma $$ photon bremsstrahlung;
- virtual QCD corrections to $q{\bar{q}}\rightarrow V\gamma$, which are needed to cancel soft-gluon singularities from (a.2) if the final-state QCD partons are allowed to become unresolved;
- $q {\bar{q}}\rightarrow Vq'{\bar{q}}'$ bremsstrahlung, which contributes at $\mathcal {O}(\alpha ^2\alpha _{\mathrm {S}})$ through the interference of $\mathcal {O}(eg_S^2)$ and $\mathcal {O}(e^3)$ tree amplitudes in the same-flavor case, $q=q'$;

Formally at $\mathcal {O}(\alpha ^2\alpha _{\mathrm {S}})$ in perturbation theory also the following contributions appear and are not included:

- $\gamma q\rightarrow V q g$ photon-induced quark bremsstrahlung,^11^ at $\mathcal {O}(\alpha ^2\alpha _{\mathrm {S}})$, which plays the dual role of NLO EW correction to the $q{\bar{q}}\rightarrow Vg$ channel and NLO QCD correction to the $\gamma q\rightarrow Vq$ channel. As discussed in Sect. 4.3, given the relatively small impact of $\gamma q\rightarrow V q$ processes at $\mathcal {O}(\alpha ^2)$, photon-induced contributions of $\mathcal {O}(\alpha _{\mathrm {S}}\alpha ^2)$ will not be included in the present study;
- real-boson emission, i.e. $pp\rightarrow VV'j$, contributes at $\mathcal {O}(\alpha ^2\alpha _{\mathrm {S}})$. As discussed in Sect. 4.5, in order to avoid double counting with diboson production, such contributions should be treated as separate background samples and not as part of the EW corrections to $pp\rightarrow Vj$.

At very high transverse momentum, EW corrections are strongly enhanced by Sudakov effects, and the inclusion of higher-order Sudakov logarithms becomes mandatory in order to achieve few-percent level accuracy. In the high-$p_\mathrm {T}$ regime, where all energy scales are far above the weak-boson mass scale, higher-order virtual EW corrections to hard scattering cross sections can be described by means of resummation formulas of the type^12^ [63, 64]

44 $$\begin{aligned} \mathrm {d}\sigma _{\mathrm {EW}}= \exp \bigg \{\int _{M_W^2}^{Q^2}\frac{\mathrm {d}t}{t} \bigg [ \int _{M_W^2}^{t}\mathrm {d}\tau \frac{\gamma (\alpha (\tau ))}{\tau } \nonumber \\ +\chi (\alpha (t))+\xi \left( \alpha (M_W^2)\right) \bigg ] \bigg \} \mathrm {d}\sigma _\mathrm {hard}, \end{aligned}$$

where $\gamma$, $\chi$ and $\xi$ are anomalous dimensions depending on the EW quantum numbers of the scattering particles. The hard cross section has the form

45 $$\begin{aligned} \mathrm {d}\sigma _\mathrm {hard}=\left[ 1+\frac{\alpha }{\pi }\delta ^{(1)}_\mathrm {hard}+\left( \frac{\alpha }{\pi }\right) ^2\delta ^{(2)}_\mathrm {hard}+\cdots \right] \mathrm {d}\sigma _{\mathrm {Born}}, \end{aligned}$$

and the correction factors $\delta ^{(k)}_\mathrm {hard}$ are finite in the limit $Q^2/M_W^2\rightarrow \infty$, while EW Sudakov and subleading high-energy logarithms of type $\alpha ^m \ln ^n\left( Q^2/M_W^2\right)$ are factorized in the exponential. Expanding in $\alpha =\alpha (M^2)$ with $\gamma _i(\alpha )=\frac{\alpha }{\pi }\gamma _i^{(1)}+\cdots \!,$ and

46 $$\begin{aligned} \alpha (t)=\alpha \left[ 1+\frac{\alpha }{\pi }b^{(1)} \ln \left( \frac{t}{M^2}\right) +\cdots \right] \end{aligned}$$

yields

47 $$\begin{aligned} \exp \bigg \{\cdots \bigg \}=1+\frac{\alpha }{\pi }\delta ^{(1)}_\mathrm {Sud}+\left( \frac{\alpha }{\pi }\right) ^2\delta ^{(2)}_\mathrm {Sud}+\cdots . \end{aligned}$$

At NLL level, which is the logarithmic accuracy at which NNLO Sudakov effects are known for $V+$ jet production [22–25, 52], the following types of logarithms are available:

48 $$\begin{aligned} \delta ^{(1)}_\mathrm {Sud}= & {} \sum _{i,j}C_{2,ij}^{(1)}\ln ^{2}\left( \frac{Q_{ij}^2}{M^2}\right) +C_{1}^{(1)}\ln ^{1}\left( \frac{Q^2}{M^2}\right) , \nonumber \\ \delta ^{(2)}_\mathrm {Sud}= & {} \sum _{i,j}C_{4,ij}^{(2)}\ln ^{4}\left( \frac{Q_{ij}^2}{M^2}\right) +C_{3}^{(2)}\ln ^{3}\left( \frac{Q^2}{M^2}\right) \nonumber \\&+\mathcal {O}\left[ \ln ^{2}\left( \frac{Q^2}{M^2}\right) \right] , \end{aligned}$$

where $M=M_W\sim M_Z$, $Q^2_{ij}=|({\hat{p}}_i\pm {\hat{p}}_j)^2|$ are the various Mandelstam invariants built from the hard momenta ${\hat{p}}_i$ of the $V+$ jet production process and $Q^2=Q_{12}^2={\hat{s}}$.

In this work we will employ the explicit NLL Sudakov results of Refs. [22–25, 52], which have been implemented, in addition to exact NLO QCD+NLO EW amplitudes, in the OpenLoops matrix-element generator [19, 40]. Let us recall that the results of Refs. [22–25, 52] are based on the high-energy limit of virtual one- and two-loop corrections regularized with a fictitious photon mass of order $M_W$. This generates logarithms of the form $\alpha ^n\ln ^k({\hat{s}}/M^2_W)$, which correspond to the combination of virtual one- and two-loop EW corrections plus corresponding photon radiation contributions up to an effective cut-off scale of order $M_W$. In the case of $V+$ jet production, for physical observables that are inclusive with respect to photon radiation, this approximation is accurate at the one-percent level [21, 22, 25].

In this work we will employ full EW results at NLO and NLL Sudakov logarithms at NNLO. In the notation of Eqs. (41)–(43), for fully differential partonic cross sections, this implies

49 $$\begin{aligned} \kappa _{\text {NLO} \,{\mathrm {EW}}}({\hat{s}}, {\hat{t}})= & {} \frac{\alpha }{\pi }\left[ \delta ^{(1)}_\mathrm {hard}+\delta ^{(1)}_\mathrm {Sud}\right] , \end{aligned}$$

50 $$\begin{aligned} \kappa _{\mathrm {NNLO}\,\mathrm {Sud}}({\hat{s}}, {\hat{t}})= & {} \left( \frac{\alpha }{\pi }\right) ^2\delta ^{(2)}_\mathrm {Sud}. \end{aligned}$$

#### Pure EW uncertainties

Assuming that the NLL Sudakov approximation at NNLO is comparably accurate as at NLO, we can consider unknown Sudakov logarithms beyond NNLO as the dominant source of EW uncertainty at high $p_\mathrm {T}$. Such Sudakov terms of relative $\mathcal {O}(\alpha ^3)$ can easily be estimated via naive exponentiation, which implies the following relations between NLO, NNLO and NNNLO terms:

51 $$\begin{aligned} \delta ^{(2)}_\mathrm {Sud}\simeq & {} \frac{1}{2}\left[ \delta ^{(1)}_\mathrm {Sud}\right] ^2,\nonumber \\ \delta ^{(3)}_\mathrm {Sud}\simeq & {} \frac{1}{3!}\left[ \delta ^{(1)}_\mathrm {Sud}\right] ^3\simeq \frac{1}{3}\delta ^{(1)}_\mathrm {Sud}\,\delta ^{(2)}_\mathrm {Sud}. \end{aligned}$$

Based on these relations, we estimate the uncertainty due to unknown high-$p_\mathrm {T}$ EW effects beyond NNLO as

52 $$\begin{aligned} \delta ^{(1)}\kappa ^{(V)}_{\mathrm {nNLO}\,{\mathrm {EW}}}(x)= & {} \frac{2}{3} \left| \kappa ^{(V)}_{\text {NLO} \,{\mathrm {EW}}}(x)\,\kappa ^{(V)}_{\mathrm {NNLO}\,\mathrm {Sud}}(x) \right| , \end{aligned}$$

which is an approximate implementation of Eq. (51), obtained by neglecting effects from angular integration, replacing $\delta ^{(1)}_\mathrm {Sud}$ by the full NLO EW correction, and multiplying the term $\delta ^{(3)}_\mathrm {Sud}$ by a factor 2, in order to be conservative.

Besides Sudakov exponentiation effects, we introduce a second source of uncertainty, defined, at nNLO EW level, as 5% of the absolute full NLO EW correction,

53 $$\begin{aligned} \delta ^{(2)} \kappa ^{(V)}_{\mathrm {nNLO}\,{\mathrm {EW}}}(x)= 0.05\, \left| \kappa ^{(V)}_{\text {NLO} \,{\mathrm {EW}}}(x)\right| . \end{aligned}$$

This type of uncertainty has a twofold motivation. At high $p_\mathrm {T}$, where Sudakov logarithms dominate, it accounts for unknown terms of order $\alpha ^2\ln ^{2}\left( \frac{Q^2}{M^2}\right)$ that can arise from effects of the form

54 $$\begin{aligned} \left( \frac{\alpha }{\pi }\right) ^2\delta ^{(1)}_\mathrm {hard}\, \delta ^{(1)}_\mathrm {Sud}= & {} \kappa _{\text {NLO} \,\mathrm {hard}}\, \kappa _{\text {NLO} \,\mathrm {Sud}}\nonumber \\\simeq & {} \kappa _{\text {NLO} \,\mathrm {hard}}\, \kappa _{\text {NLO} \,{\mathrm {EW}}}. \end{aligned}$$

**Fig. 9:**
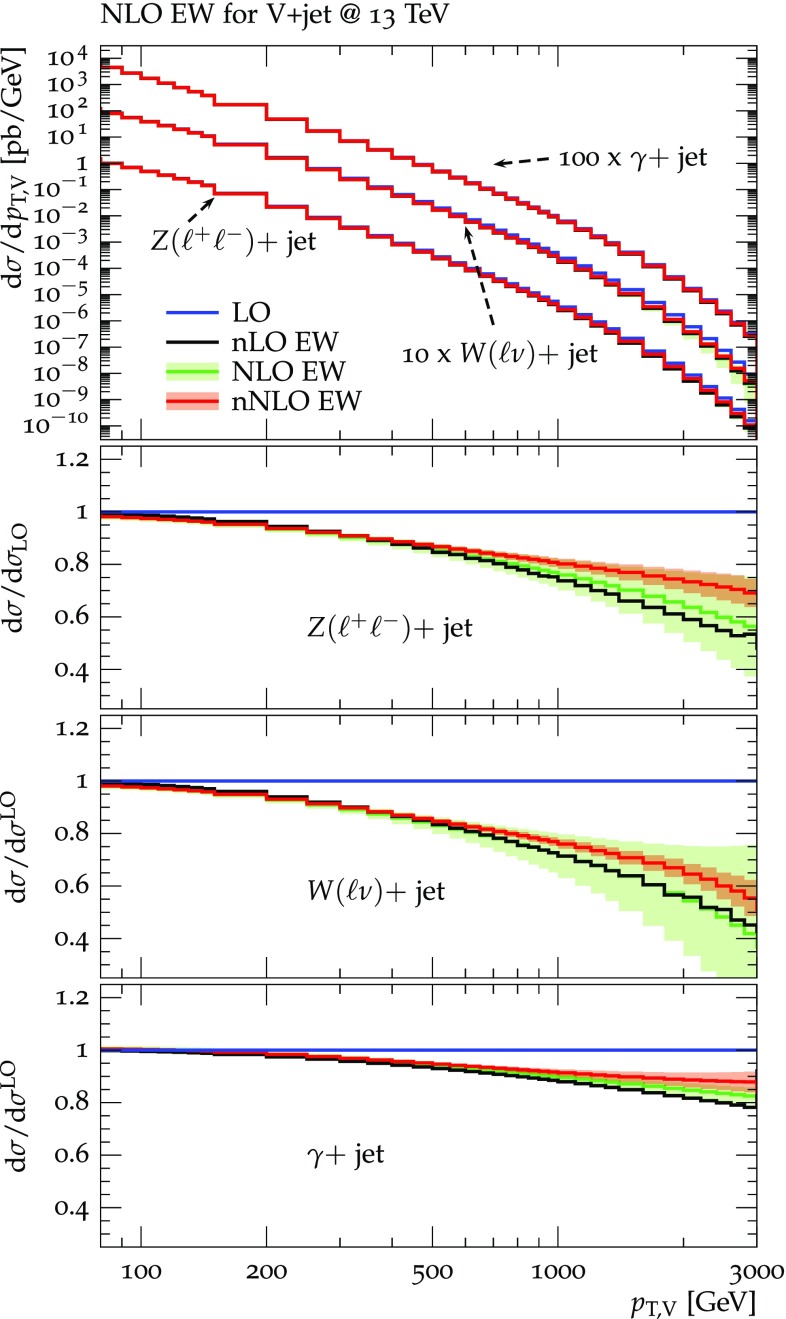
Higher-order EW predictions and uncertainties for different $pp\rightarrow V+$ jet processes at 13 TeV. The main frame displays absolute predictions at LO (blue), NLO EW (green) and nNLO EW (red), as well as NLL Sudakov logarithms at NLO (black), which are denoted as nLO EW. In the ratio plots all results are normalized to LO. Uncertainties at nNLO EW (red band) are evaluated by combining in quadrature the corresponding variations $\delta ^{(i)}\kappa ^{(V)}_{\mathrm {nNLO}\,{\mathrm {EW}}}$ as defined in Eqs. (52), (53) and (56) and for $\delta ^{(i)}\kappa ^{(V)}_{\text {NLO} \,{\mathrm {EW}}}$ in Eq. (57)

In general, the non-Sudakov factor $\kappa _{\text {NLO} \,\mathrm {hard}}=(\frac{\alpha }{\pi })\delta ^{(1)}_\mathrm {hard}$ can amount to several percent, e.g. due to photon- bremsstrahlung effects in highly exclusive observables. However, for the boson-$p_\mathrm {T}$ distributions considered in this paper, where dressed leptons are used, the quality of the Sudakov approximation observed in Fig. 9 indicates that $\kappa _{\text {NLO} \,\mathrm {hard}}$ is very small. Nevertheless, to be conservative, in Eq. (53) we choose a prefactor that allows for effects as large as $\kappa _{\text {NLO} \,\mathrm {hard}}=5\%$.

As a second motivation, the uncertainty (53) accounts also for NNLO effects of type $\left( \frac{\alpha }{\pi }\right) ^2\delta ^{(2)}_\mathrm {hard}$, which can become relevant in the case where hard contributions dominate. In this situation, Eq. (53) amounts to a bound on hard NNLO effects,

55 $$\begin{aligned} \left( \frac{\alpha }{\pi }\right) ^2\delta ^{(2)}_\mathrm {hard}\le 0.05\, \kappa _{\text {NLO} \,{\mathrm {EW}}} \simeq 0.05 \left( \frac{\alpha }{\pi }\right) \delta ^{(1)}_\mathrm {hard}, \end{aligned}$$

which corresponds to $\delta ^{(2)}_\mathrm {hard}\le \frac{0.05\pi }{\alpha }\delta ^{(1)}_\mathrm {hard}\simeq 20\,\delta ^{(1)}_\mathrm {hard}$. This limit should be conservative enough to hold also in situations where the NLO hard correction is accidentally small with respect to its NNLO counterpart.

In order to account for the limitations of the Sudakov approximation at nNLO in a sufficiently conservative way, we introduce an additional source of uncertainty defined as the difference between the rigorous NLL Sudakov approximation (50) and a naive exponentiation of the full NLO EW correction,

56 $$\begin{aligned} \delta ^{(3)}\kappa ^{(V)}_{\mathrm {nNLO}\,{\mathrm {EW}}}(x)= & {} \left| \kappa ^{(V)}_{\mathrm {NNLO}\,\mathrm {Sud}}(x)- \frac{1}{2}[\kappa ^{(V)}_{\text {NLO} \,{\mathrm {EW}}}(x)]^2 \right| .\nonumber \\ \end{aligned}$$

This expression provides an estimate of the typical size of terms of type $\left[ \delta ^{(1)}_\mathrm {hard}\right] ^2$ and $\delta ^{(1)}_\mathrm {hard}\times \delta ^{(1)}_\mathrm {Sud}$.

In correspondence to the nNLO uncertainties of Eqs. (52), (53) and (56), at NLO EW we introduce uncertainties $\delta ^{(i)}\kappa _{\text {NLO} \,{\mathrm {EW}}}$, defined as

57 $$\begin{aligned} \delta ^{(1)}\kappa ^{(V)}_{\text {NLO} \,{\mathrm {EW}}}(x)= & {} \frac{2}{2} \left[ \kappa ^{(V)}_{\text {NLO} \,{\mathrm {EW}}}(x)\right] ^2,\nonumber \\ \delta ^{(2)}\kappa ^{(V)}_{\text {NLO} \,{\mathrm {EW}}}(x)= & {} 2000\times \left( \frac{\alpha }{\pi }\right) ^2 \simeq 1.2\% ,\nonumber \\ \delta ^{(3)}\kappa ^{(V)}_{\text {NLO} \,{\mathrm {EW}}}(x)= & {} 0. \end{aligned}$$

Here the first term is the direct transposition of Eq. (52) to NLO. It accounts for the unknown $\mathcal {O}{(\alpha ^2)}$ Sudakov terms $\delta _\mathrm {Sud}^{(2)}$ in Eq. (51) supplemented with an extra factor of 2. As explained in the following, the second uncertainty in Eq. (57) is the NLO counterpart of the nNLO EW uncertainty (53). The latter accounts for unknown $\mathcal {O}{(\alpha ^2)}$ terms of type (54) and (55), which correspond to the intrinsic uncertainty of the employed Sudakov approximation at nNLO. At NLO EW the situation is different, since the calculations are exact, i.e. there are no unknown terms of $\mathcal {O}(\alpha )$. Thus, we assume an uncertainty $\delta ^{(2)}\kappa ^{(V)}_{\text {NLO} \,{\mathrm {EW}}}(x)$ of type $\left( \frac{\alpha }{\pi }\right) ^2\delta ^{(2)}_\mathrm {hard}$. We do not consider additional uncertainties of type $\left( \frac{\alpha }{\pi }\right) ^2\delta ^{(2)}_\mathrm {Sud}$ since they are already covered by the first term in Eq. (57). As estimate of the size of the unknown $\delta ^{(2)}_\mathrm {hard}$ coefficient, following the discussion of Eq. (55), we impose a very generous upper bound to the ratio between $\delta ^{(2)}_\mathrm {hard}$ and $\delta ^{(1)}_\mathrm {hard}$. To be conservative, at NLO EW we adopt a ten times looser bound as compared to nNLO EW, i.e. we require $\delta ^{(2)}_\mathrm {hard}\lesssim 200\,\delta ^{(1)}_\mathrm {hard}$. Finally, setting $\delta ^{(1)}_\mathrm {hard}=10$, which corresponds to the typical size of non-Sudakov-enhanced EW corrections, $10\times \left( \frac{\alpha }{\pi }\right) \simeq 2\%$, we arrive at $2000\times \left( \frac{\alpha }{\pi }\right) ^2$ for the second term in Eq. (57). The third uncertainty in Eq. (57) is set to zero, since there is no counterpart of Eq. (56) at NLO.

Similarly as for QCD uncertainties, the EW uncertainties in Eqs. (52), (53), (56) and (57), can be parametrized in terms of nuisance parameters ${\varvec{\varepsilon }}_{\mathrm {EW}}$ and combined via

58 $$\begin{aligned} \frac{\mathrm{d}}{\mathrm{d}x}\sigma ^{(V)}_{{\mathrm {EW}}}({\varvec{\varepsilon }}_{\mathrm {EW}},{\mathbf {\varepsilon }}_{\mathrm {QCD}})= & {} \left[ \kappa ^{(V)}_{{\mathrm {EW}}}(x) +\sum _{i=1}^3\varepsilon ^{(V)}_{{\mathrm {EW}},i}\,\delta ^{(i)}\kappa ^{(V)}_{\mathrm {EW}}(x)\right] \nonumber \\&\times \frac{\mathrm{d}}{\mathrm{d}x}\sigma ^{(V)}_{\text {LO} \,{\mathrm {QCD}}}({\varvec{\varepsilon }}_{\mathrm {QCD}}), \end{aligned}$$

where EW stands for NLO EW or nNLO EW. The nuisance parameters $\varepsilon ^{(V)}_{{\mathrm {EW}},i}$ should be Gaussian distributed with one standard deviation corresponding to the range $\varepsilon ^{(V)}_{{\mathrm {EW}},i}\in [-1,+1]$, and their variations should be applied in a correlated way across $p_\mathrm {T}$-bins. Since the first uncertainty (52) reflects the universal exponentiation properties of Sudakov EW corrections, which permits to predict the magnitude and size of the dominant higher-order corrections for each individual processes, this variation should be correlated across processes, i.e. a single nuisance parameter should be used,

59 $$\begin{aligned} \varepsilon ^{(W^\pm )}_{{\mathrm {EW}},1}= \varepsilon ^{(Z)}_{{\mathrm {EW}},1}= \varepsilon ^{(\gamma )}_{{\mathrm {EW}},1}= \varepsilon _{{\mathrm {EW}},1}. \end{aligned}$$

In contrast, the remaining EW uncertainties (53) and (56) describe subleading NNLO effects whose sign, magnitude and process dependence are unknown. Thus these uncertainties should be treated as uncorrelated; i.e. independent nuisance parameters $\varepsilon ^{(V)}_{{\mathrm {EW}},2}$ and $\varepsilon ^{(V)}_{{\mathrm {EW}},3}$ should be used for each process.

#### Numerical results

**Fig. 10:**
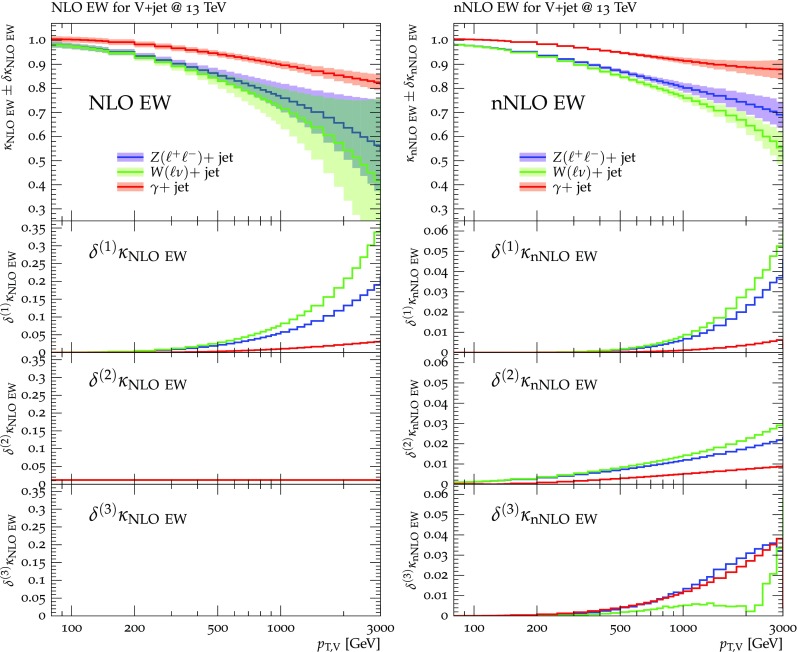
NLO EW (left) and nNLO EW (right) $\kappa$-factors for the various $pp\rightarrow V+$ jet processes at 13 TeV. The individual uncertainties $\delta ^{(i)}\kappa ^{(V)}_{{\mathrm {EW}}}$ are defined in Eqs. (52), (53) and (56), at nNLO and in Eq. (57) at NLO. The bands in the main frame correspond to their combination in quadrature

**Fig. 11:**
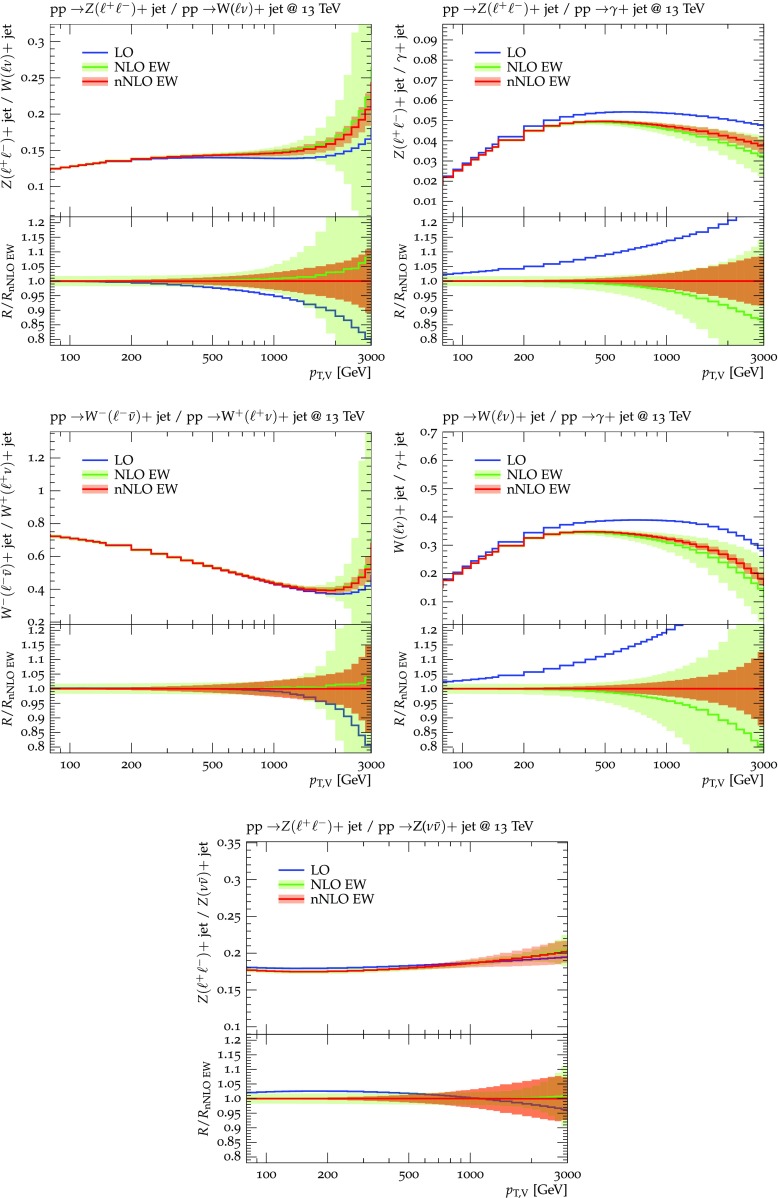
Ratios of $p_\mathrm {T}$-distributions for various $pp\rightarrow V+$jet processes at LO, NLO EW and nNLO EW accuracy. Relative uncertainties normalized to nNLO EW are illustrated in the lower frames. The bands correspond to a combination (in quadrature) of the three EW uncertainties $\delta ^{(i)}\kappa ^{(V)}_{{\mathrm {EW}}}$ defined in Eqs. (52), (53) and (56) at nNLO and in Eq. (57) at NLO. As discussed in the text, the uncertainty $\delta ^{(1)}\kappa ^{(V)}_{{\mathrm {EW}}}$ is correlated amongst processes, while the effect of $\delta ^{(2)}\kappa ^{(V)}_{{\mathrm {EW}}}$ and $\delta ^{(3)}\kappa ^{(V)}_{{\mathrm {EW}}}$ in the numerator and denominator of ratios is kept uncorrelated, i.e. added in quadrature

Predictions for $V+$jet distributions and their ratios at LO, $\text {NLO} \,{\mathrm {EW}}$ and $\mathrm {nNLO}\,{\mathrm {EW}}$ are presented in Figs. 9, 10 and 11 as well as in Figs. 20 and 21 (see Appendix B). In Figs. 10, 20 and 21, the EW uncertainties defined in Eqs. (52), (53), and (56) are shown separately, while in Figs. 9 and 11 they are combined in quadrature.

Contrary to the case of QCD corrections, higher-order EW effects have a significant impact on the shapes of $p_{\mathrm {T}}$ distributions as well as a pronounced dependence on the scattering process. This behavior is mainly due to the $p_{\mathrm {T}}$ dependence of EW Sudakov logarithms and their dependence on the SU(2) charges of the produced vector bosons.

As can be seen in Fig. 9, the vector-boson $p_{\mathrm {T}}$ spectra receive negative EW corrections that grow with $p_{\mathrm {T}}$ and become very sizable in the tails. At the TeV scale, NLO EW effects reach 20–50% for $Z+$jet and $W+$jet production, and 10–15% for $\gamma +$jet production. As expected from exponentiation, NNLO Sudakov logarithms have positive sign. Thus they compensate in part for the impact of NLO EW corrections.

In Fig. 9 exact NLO EW results are also compared to the NLL Sudakov approximation at the same order, denoted $\mathrm {nLO}\,{\mathrm {EW}}$. The observed agreement indicates that the Sudakov approximation at NLO works very well, thereby supporting the usage of EW Sudakov logarithms at $\mathrm {NNLO}$. Moreover, the fact that $\mathrm {nNLO}\,{\mathrm {EW}}$ results are well consistent with NLO predictions supplemented by the corresponding uncertainties (57) provides an important confirmation of the goodness of the proposed approach for the estimate of EW uncertainties.

The importance of NLO and $\mathrm {nNLO}\,{\mathrm {EW}}$ corrections for different processes and the role of individual uncertainties is shown in more detail in Fig. 10. Regarding the size of EW uncertainties we observe that the inclusion of $\mathrm {nNLO}\,{\mathrm {EW}}$ corrections is crucial in order to achieve few-percent accuracy in the tails, while uncertainties at $\text {NLO} \,{\mathrm {EW}}$ can be as large as 10% or beyond.

As shown in Fig. 11, the various ratios of $p_{\mathrm {T}}$ distributions and their shape receive significant EW corrections, with the largest effects observed in the $Z(\ell ^+\ell ^-)/\gamma$ and $W/\gamma$ ratios. In these ratios the remaining combined EW uncertainties are at the level of few percent in the TeV range, reaching about $5\%$ for $p_{\mathrm{T}, V}\simeq 2$ TeV. Interestingly, also the $Z(\ell ^+\ell ^-)/Z(\nu {\bar{\nu }})$ and $W^-/W^+$ ratios receive non-negligible EW corrections. In the case of the $W^-/W^+$ ratio this is due to the behavior of mixed QCD–EW interference contributions at high $p_{\mathrm {T}}$, which yield relevant (negative) contributions in $W^{+}+$jet production but less in $W^{-}+$jet production. As for the $Z(\ell ^+\ell ^-)/Z(\nu {\bar{\nu }})$ ratio, the observed EW effects can be attributed to $p_{\mathrm {T}}$-migration effects induced by QED radiation off leptons. At moderate $p_{\mathrm {T},Z}$, the invariant mass of photon-lepton pairs that lie inside the recombination cone $\varDelta R_{\ell \gamma }<0.1$ is well below $M_Z$. Thus a significant fraction of the $Z\rightarrow \ell ^+\ell ^-\gamma$ phase space does not undergo photon-lepton recombination, and photon radiation results in a negative mass and momentum shift for the $\ell ^+\ell ^-$ system. The *Z*-mass shift is typically not sufficient to push $Z\rightarrow \ell ^+\ell ^-\gamma$ events outside the inclusive $m_{\ell \ell }$ window defined in Sect. 3.2. However, the reduction of the reconstructed $p_{\mathrm {T},\ell \ell }$ results in a negative correction to the $Z(\ell ^+\ell ^-)/Z(\nu {\bar{\nu }})$ ratio. Vice versa, for $p_{\mathrm {T},Z}\gtrsim 1$ TeV the recombination cone $\varDelta R_{\ell \gamma }<0.1$ covers photon-lepton invariant masses up to $p_{\mathrm {T},Z}\varDelta _{\ell \gamma }> M_Z$, i.e. beyond the $Z\rightarrow \ell ^+\ell ^-\gamma$ phase space. As a result, $p_{\mathrm {T},\ell \ell }$ starts capturing a non-negligible amount of ISR QED radiation, which results in a positive shift of $p_{\mathrm {T},\ell \ell }$ and thus in a positive correction to the $Z(\ell ^+\ell ^-)/Z(\nu {\bar{\nu }})$ ratio. Note that the quantitative impact of such corrections depends on the choice of the $m_{\ell \ell }$ mass window. Thus, for a consistent implementation of the predictions presented in this study it is crucial to reweight MC samples using the $m_{\ell \ell }$ window defined in Sect. 3.2. Moreover, in order to guarantee a consistent extrapolation of QED radiative effects to the $m_{\ell \ell }$ window employed in experimental analyses, it is mandatory to employ MC samples that account for QED radiation off leptons.

### Photon-induced production and QED effects on PDFs

Higher-order QCD and EW calculations for $pp\rightarrow V+$ jet require PDFs at a corresponding accuracy level, i.e. including also QED corrections. The effect of QED interactions on parton densities is twofold. Firstly they introduce a photon parton distribution and so open up partonic channels such as $\gamma q \rightarrow V q'$. Secondly they modify the quark (and even gluon) PDFs both through QED effects in the initial conditions and especially in the DGLAP evolution.

Photon-induced $V+$ jet production is accounted for by the term $\frac{\mathrm{d}}{\mathrm{d}x}\sigma ^{(V)}_{\gamma -\mathrm{ind.}}$ in Eq. (25). It might become relevant in the TeV range, especially in the case of $W+$ jet production [19, 20], where the initial-state photon directly couples to a virtual *W* boson in the *t*-channel. Such contributions are suppressed by a relative factor $\alpha /\alpha _S$ and can be treated at LO, which corresponds to $\gamma q\rightarrow Vq$ at $\mathcal {O}(\alpha ^2)$ or, if necessary, at NLO QCD, i.e. up to order $\mathcal {O}(\alpha ^2\alpha _{\mathrm {S}})$. This order comprises:

1. virtual QCD corrections to $\gamma q\rightarrow Vq$;
2. $$\gamma g\rightarrow Vq{\bar{q}}$$ quark bremsstrahlung;
3. $$\gamma q\rightarrow Vqg$$ gluon bremsstrahlung.

The latter can also be understood as photon-induced quark-bremsstrahlung NLO EW contribution to the dominant $q{\bar{q}}$ channel. See the contributions of type (a.5) in Sect. 4.2.

**Fig. 12:**
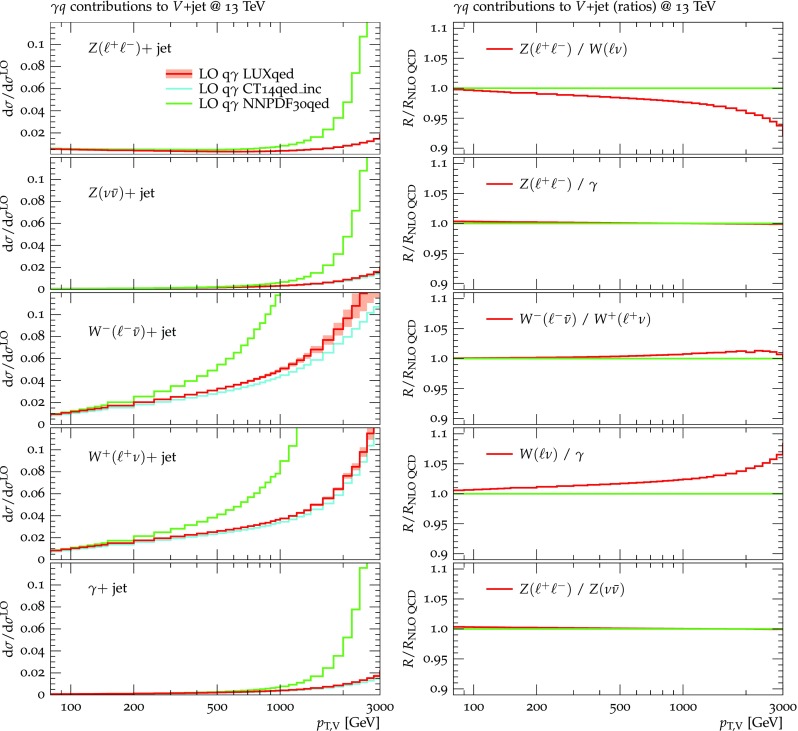
The left plot illustrates the impact of photon-induced contributions at LO, i.e. $\gamma p\rightarrow V+$ jet at $\mathcal {O}(\alpha ^2)$, relative to $pp\rightarrow V+$ jet at LO QCD for different $V+\,$jet processes. Predictions obtained with LUXqed_plus_PDF4LHC15_nnlo_100, CT14qed_inc and NNPDF30qed PDFs are compared. The error band, shown only for the LUXqed_plus_PDF4LHC15_nnlo_100 prediction, reflects PDF uncertainties. The right plot shows ratios of $V+$jet distributions at NLO QCD with (red) and without (green) $\gamma$-induced contributions based on LUXqed_plus_PDF4LHC15_nnlo_100 PDFs

Figure 12 illustrates the impact of photon-induced $V+$ jet production at LO according to three recent PDF sets that implement QED corrections. Effects of the order of 5–10% for $W+$jet can be observed in the TeV region if CT14qed_inc [65] or LUXqed PDFs [39] are used. Much larger effects are found with NNPDF30qed [66, 67]. The impact of photon-induced production to $Z+$jet (and also $\gamma +$jet) processes on the other hand is negligible [17, 18].

For the description of PDFs and their uncertainties we will use the LUXqed PDFs and their intrinsic uncertainties, given that this set of parton distributions implements a model-independent, data-driven determination of the photon distribution. From Fig. 12 one sees that the LUXqed uncertainties for $\gamma p\rightarrow V+$ jet are small. Using the CT14qed_inc PDFs, based on a non-perturbative model with limited data-based constraints for the inelastic contribution, would result in fairly similar photon-induced cross sections but somewhat larger uncertainties (not shown) as compared to LUXqed PDFs. The NNPDF30qed parton distributions are model independent and data driven, but they are based on a different approach from LUXqed for deducing the photon distribution from data, which results in large uncertainties in the photon-induced component, of the order of $100\%$ for $pp\rightarrow \ell ^+\nu _\ell +$ jet at $p_{\mathrm {T},\ell }=1\,$TeV [20].

We have verified that the NLO QCD corrections to photon-induced production have an impact at the percent level relative to $\mathcal {O}(\alpha ^2)$ and can safely be omitted. This implies that $\gamma p\rightarrow V+$ jet can be regarded as independent processes. Thus photon-induced $V+$ jet production can be either included through the parton-level predictions provided in this study or handled as separate background processes through dedicated MC simulations.

**Fig. 13:**
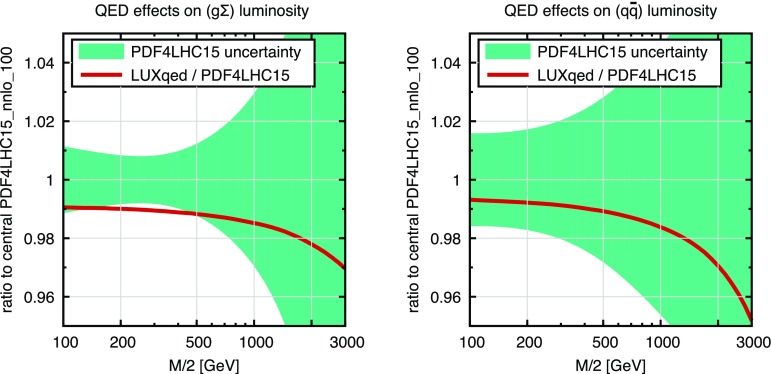
Impact of QED effects on the two partonic luminosities ($g\Sigma$ and $q{\bar{q}}$) that contribute dominantly to the $Z+$jet cross section. The luminosity for producing a system of mass *M* from two flavors *a* and *b* is defined as $\mathcal{L}_{ab} = \int _{M^2\!/\!s}^1 \frac{dx}{x} f_{a/p}(x, M^2) f_{b/p}(\frac{M^2}{xs}, M^2)$ and the $g\Sigma$ luminosity corresponds to $2 \sum _i (\mathcal{L}_{gq_i} + \mathcal{L}_{g{\bar{q}}_i})$, while the $q{\bar{q}}$ luminosity corresponds to $2\sum _i \mathcal{L}_{q_i{\bar{q}}_i}$, where *i* runs over quark flavors. The solid red lines correspond to the ratio of luminosities obtained with the LUXqed_plus_PDF4LHC15_nnlo_100 [39] and PDF4LHC15_nnlo_100 [33] sets, where a given *M* / 2 value corresponds roughly to the same $p_{\mathrm {T},Z}$. The bands represent the PDF4LHC15_nnlo_100 uncertainty, shown for comparison

Concerning the size of the QED effects on the QCD partons, Fig. 13 examines the two main parton luminosities that contribute to the $Z+$jet process, i.e. $g\Sigma =2 \sum _i (\mathcal{L}_{gq_i} + \mathcal{L}_{g{\bar{q}}_i})$ (which dominates) and $q{\bar{q}}=2\sum _i \mathcal{L}_{q_i{\bar{q}}_i}$ (which accounts for the remaining $15\%{-}30\%$). It shows the ratio of these luminosities in LUXqed_plus_PDF4LHC15_nnlo relative to the PDF4LHC15_nnlo set on which it is based. The ratio is given as a function of half the partonic invariant mass, *M* / 2, which is commensurate with the $p_{\mathrm {T}}$ of the *Z*.

Most of the difference between the LUXqed set and PDF4LHC15_nnlo results in Fig. 13 comes from the QED effects in the DGLAP evolution [68], with photon emission during the evolution reducing the momentum in the quarks. This effect reaches about $2\%$ at $2\,$TeV for the $g\Sigma$ luminosity. There is also a part of the correction associated with the impact of QED effects on the initial partons. In the LUXqed set this has been approximated by absorbing the photon momentum from the gluon distribution in PDF4LHC15_nnlo and keeping the quarks unchanged at a scale of $10\,\text {GeV}$. This is an ad hoc procedure, however, insofar as the photon carries only $\simeq 0.3\%$ of the proton momentum (at a scale of $10\,\text {GeV}$), the uncertainty associated with the arbitrariness of this choice should be below $1\%$.

### PDF uncertainties

**Fig. 14:**
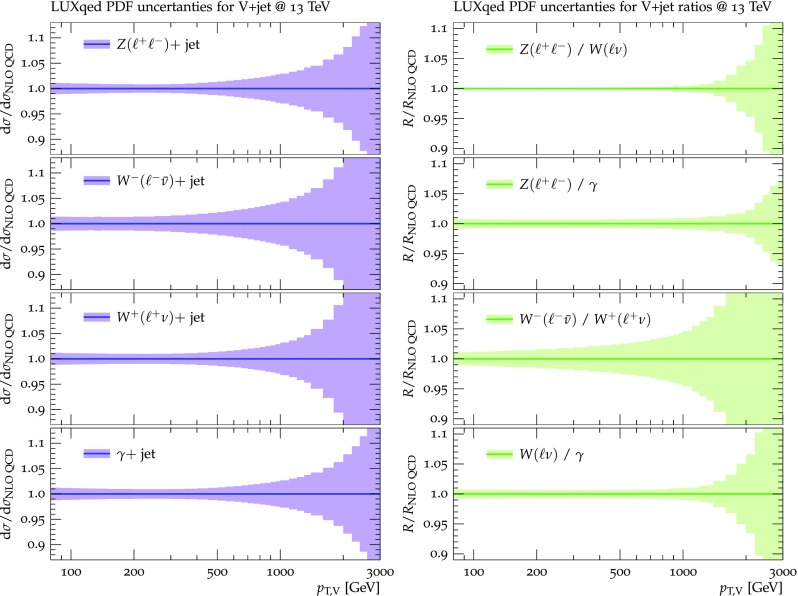
Relative LUXqed_plus_PDF4LHC15_nnlo_100 PDF uncertainties on the nominal $p_{\mathrm {T}}$ distributions for the different $pp\rightarrow V+$jet processes at 13 TeV evaluated to NLO QCD are shown on the left. Corresponding PDF uncertainties for ratios of $V+$ jet distributions are shown on the right. In the ratios different PDF replicas are correlated across processes and the resulting errors on the respective ratio are combined in quadrature

The role of PDF uncertainties can be significant especially at high-$p_\mathrm {T}$, where PDFs tend to be less precise. In Fig. 14 we illustrate the effect of PDF uncertainties within LUXqed (for the quark and gluon uncertainties based on PDF4LHC15_nnlo_100) for the different $V+$jets processes and process ratios at NLO QCD. Up to about 800 GeV the PDF uncertainties on the nominal $p_{\mathrm {T}}$ distributions remain below $2\%$. In the tails of the distributions the PDF uncertainties significantly increase. They grow beyond $5\%$ for $p_{\mathrm {T}} \gtrsim 1.5$ TeV. In the *Z* / *W* ratio the PDF uncertainties cancel almost completely and remain below $0.5(2)\%$ up to $p_{\mathrm {T}} \approx 800(1500)$ GeV. In the $Z/\gamma$ and $W/\gamma$ ratios the PDF uncertainties are at the level of 1–$2\%$ up to $p_{\mathrm {T}} \approx 1300$ GeV, while the $W^-/W^+$ ratio is subject to PDF uncertainties beyond $5\%$ already for $p_{\mathrm {T}} \gtrsim 1$ TeV, driven by uncertainties on the *u* / *d* ratio at large Bjorken-*x* [3].

To keep track of PDF uncertainties in the combination of QCD and EW corrections we introduce a generalized set of QCD nuisance parameters,

60 $$\begin{aligned} {\varvec{\varepsilon }}_{\mathrm {QCD}}=\left( \varepsilon _{{\mathrm {QCD}},1},\varepsilon _{{\mathrm {QCD}},2},\varepsilon _{{\mathrm {QCD}},3}, \varepsilon _{\mathrm {PDF},1},\varepsilon _{\mathrm {PDF},2},\ldots \right) , \end{aligned}$$

which comprises QCD scale and shape variations, as well as process-correlation and PDF uncertainties. To this end we extend Eq. (39),

61 $$\begin{aligned}&\frac{\mathrm{d}}{\mathrm{d}x}\sigma ^{(V)}_{\mathrm {N}^k\mathrm {LO}\,{\mathrm {QCD}}}({\varvec{\varepsilon }}_{{\mathrm {QCD}}})= \left[ K^{(V)}_{\mathrm {N}^k\mathrm {LO}}(x) \right. \nonumber \\&\quad \left. +\sum _{i=1}^3\varepsilon _{{\mathrm {QCD}},i}\,\delta ^{(i)} K^{(V)}_{\mathrm {N}^k\mathrm {LO}}(x) + \sum _{i=1}^{107}\varepsilon _{\mathrm {PDF},i}\,\delta ^{(i)} K^{(V)}_{\mathrm {PDF}}(x) \right] \nonumber \\&\quad \times \,\frac{\mathrm{d}}{\mathrm{d}x}\sigma ^{(V)}_{\text {LO} \,{\mathrm {QCD}}}({\varvec{\mu }}_0)\,, \end{aligned}$$

introducing a sum over the 107 independent Hessian PDF replicas provided by the PDF set LUXqed_plus_PDF4LHC15_nnlo. Such a combination corresponds to the PDF4LHC recommendation as detailed in Eq. (20) of Ref. [33]. These PDF variations should be applied in a fully correlated way across processes and $p_\mathrm {T}$ bins. As specified in more detail in Sect. 4.6, the various uncertainties parametrized through Eq. (60) should be applied at the level of QCD calculations and treated on the same footing in the combination of QCD and EW corrections.

### Real-boson emission

Inclusive diboson production (in particular $pp\rightarrow VV'+$jets) can be understood as the real-emission counterpart to NLO EW corrections to $pp\rightarrow V+$ jet. Both contributions are separately finite and well defined if $V'=W,Z$. Although they are expected to cancel against each other to a certain (typically small) extent, in practice one should only make sure that both types of processes, $pp\rightarrow V+$ jet and $pp\rightarrow VV'$(+jets) with leptonic and hadronic decays of the $V'$, are included in the analysis, and, in order to avoid double counting, contributions of type $VV'$(+jets) should be included in separate diboson MC samples and not as EW correction effects in $V+$ jets samples. Unless a very strong cancellation is observed (which is typically not the case), there is no reason to worry about the possible correlation of uncertainties in $V+$ jets and $VV'$(+jets) production, i.e. one can treat the respective uncertainties as uncorrelated.

As concerns the accuracy of MC simulations of $pp\rightarrow VV'$(+jets), it is important to notice that a large diboson background to inclusive vector-boson production at high $p_\mathrm {T}$ is expected to arise from $pp\rightarrow VV'j$ topologies with a hard back-to-back *Vj* system accompanied by a relatively soft extra vector boson. This calls for a reliable description of $VV'+$ jet including QCD (and possibly EW) corrections. Thus we recommend the use of merged diboson samples that include at least one extra jet at matrix-element level. At the TeV scale, the EW corrections to $pp\rightarrow VV'+$ jet can become quite large [69, 70] and should ultimately be included, together with the corresponding QCD corrections [71–78].

### Combination of QCD and electroweak corrections

The combination (25) of higher-order predictions presented in the previous sections can be cast in the form

62 $$\begin{aligned} \frac{\mathrm{d}}{\mathrm{d}x}\sigma ^{(V)}_{\mathrm {TH}}({\varvec{\mu }})= & {} K^{(V)}_{\mathrm {TH}}(x,{\varvec{\mu }})\, \frac{\mathrm{d}}{\mathrm{d}x}\sigma ^{(V)}_{\text {LO} \,{\mathrm {QCD}}}({\varvec{\mu }}_0) \nonumber \\&+\frac{\mathrm{d}}{\mathrm{d}x}\sigma ^{(V)}_{\gamma -\mathrm{ind.}} (x,{\varvec{\mu }}), \end{aligned}$$

where

63 $$\begin{aligned} K^{(V)}_{\mathrm {TH}} = K^{(V)}_{\mathrm {TH},\oplus }(x,{\varvec{\mu }})= & {} K_{\mathrm {N}^k\mathrm {LO}}^{(V)}(x,{\varvec{\mu }})\nonumber \\&+ \kappa _{{\mathrm {EW}}}^{(V)}(x)\, K_{\text {LO}}^{(V)}(x,{\varvec{\mu }}) \end{aligned}$$

corresponds to the standard additive combination of QCD and EW corrections as defined, respectively, in Eqs. (27) and (41)–(43). Note that the scale-dependent LO QCD *K*-factor in Eq. (63) is due to the fact that QCD and EW correction factors are normalized to $\sigma _{\text {LO} \,{\mathrm {QCD}}}^{(V)}({\varvec{\mu }}_0)$ and $\sigma _{\text {LO} \,{\mathrm {QCD}}}^{(V)}({\varvec{\mu }})$, respectively.

Mixed QCD–EW corrections of relative $\mathcal {O}(\alpha \alpha _{\mathrm {S}})$ are not known to date. However, it is possible to obtain an improved prediction that partially includes such mixed effects by combining higher-order EW and QCD corrections through a factorized prescription,^13^

64 $$\begin{aligned} K^{(V)}_{\mathrm {TH}}= K^{(V)}_{\mathrm {TH},\otimes }(x,{\varvec{\mu }}) = K_{\mathrm {N}^k\mathrm {LO}}^{(V)}(x,{\varvec{\mu }})\left[ 1 + \kappa _{{\mathrm {EW}}}^{(V)}(x)\right] . \end{aligned}$$

The higher-order terms induced by this factorized formula can be written as

65 $$\begin{aligned} K^{(V)}_{\mathrm {TH},\otimes }(x,{\varvec{\mu }})-K^{(V)}_{\mathrm {TH},\oplus }(x,{\varvec{\mu }})= & {} \kappa _{\mathrm {N}^k\mathrm {LO}}^{(V)}(x,{\varvec{\mu }})\,\kappa _{{\mathrm {EW}}}^{(V)}(x), \end{aligned}$$

where $\kappa _{\mathrm {N}^k\mathrm {LO}}^{(V)}$ denotes the pure higher-order contribution to the QCD *K*-factor, i.e.

66 $$\begin{aligned} K_{\mathrm {N}^k\mathrm {LO}}^{(V)}(x,{\varvec{\mu }}) = K_{\text {LO}}^{(V)}(x,{\varvec{\mu }}) + \kappa _{\mathrm {N}^k\mathrm {LO}}^{(V)}(x,{\varvec{\mu }}), \end{aligned}$$

in analogy with the definition of the $\kappa _{{\mathrm {EW}}}$ correction factor (41).

The prescription (64) is motivated by the factorization of QCD corrections from the large Sudakov-enhanced EW corrections at high energies [64] and by the observation that in cases where the multiplicative and additive approach are far apart from each other, such as in the presence of giant *K*-factors [19, 81], the former turns out to be much more reliable. In general, when QCD and EW corrections are simultaneously enhanced, the $\mathcal {O}(\alpha \alpha _{\mathrm {S}})$ mixed terms that are controlled by the multiplicative prescription can become quite significant. We also note that, thanks to the fact that the relative EW correction factors $\kappa _{{\mathrm {EW}}}^{(V)}(x)$ are essentially insensitive to QCD scale variations, the scale dependence of the multiplicative combination (64) is similar to pure $\mathrm {N}^k\mathrm {LO}$ QCD predictions. In contrast, the additive approach (63) can suffer from sizable scale uncertainties when EW corrections become large.

In order to estimate the typical size of higher-order effects that are not captured by the factorized prescription (64), we cast mixed QCD–EW corrections of $\mathcal {O}(\alpha \alpha _{\mathrm {S}})$ in the form

67 $$\begin{aligned} K^{(V)}_{{\mathrm {mix}}}(x,{\varvec{\mu }})= & {} \frac{\frac{\mathrm{d}}{\mathrm{d}x}\varDelta \sigma _{{\mathrm {mix}}}^{(V)}(x,{\varvec{\mu }})}{\frac{\mathrm{d}}{\mathrm{d}x}\sigma _{\text {LO}}^{(V)}(x,{\varvec{\mu }}_0)} \nonumber \\= & {} \kappa _{\mathrm {N}^k\mathrm {LO}}^{(V)}(x,{\varvec{\mu }})\left[ \kappa _{{\mathrm {EW}}}^{(V)}(x)+ \delta \kappa ^{(V)}_{{\mathrm {mix}}}(x) \right] , \end{aligned}$$

and to model the non-factorizing term we use the simple Ansatz^14^

68 $$\begin{aligned} \delta \kappa ^{(V)}_{{\mathrm {mix}}}(x)= & {} \xi ^{(V)}\, \kappa _{{\mathrm {EW}}}^{(V)}(x). \end{aligned}$$

The expectation that the bulk of QCD and EW corrections factorize implies that the absolute value of the free process-dependent factors $\xi ^{(V)}$ should be well below 1. Note that Eq. (68) is equivalent to

69 $$\begin{aligned} \delta K^{(V)}_{{\mathrm {mix}}}(x,{\varvec{\mu }})= & {} \xi ^{(V)} \left[ K^{(V)}_{\mathrm {TH},\otimes }(x,{\varvec{\mu }}) - K^{(V)}_{\mathrm {TH},\oplus }(x,{\varvec{\mu }}) \right] , \end{aligned}$$

i.e. we assume that non-factorizing EW–QCD mixed terms are proportional to the difference between the additive and multiplicative combination of QCD and EW corrections.

The NLO EW corrections to $pp\rightarrow V+2$ jets [19, 51], which represent a real–virtual contribution to the unknown mixed EW–QCD NNLO corrections to $V+$ jet production, can provide useful insights into the typical size of the $\xi ^{(V)}$ factors and the goodness of the Ansatz (67)–(68). In particular, starting from the $\mathcal {O}(\alpha \alpha _{\mathrm {S}})$ contributions to Eq. (67),

70 $$\begin{aligned} K^{(V)}_{\mathrm {NNLO}\, {\mathrm {mix}}}(x,{\varvec{\mu }})= & {} \kappa _{\text {NLO}}^{(V)}(x,{\varvec{\mu }}) \left[ \kappa _{\text {NLO} \, {\mathrm {EW}}}^{(V)}(x) \right. \nonumber \\&+ \left. \delta \kappa ^{(V)}_{\mathrm {NNLO}\,{\mathrm {mix}}}(x)\right] , \end{aligned}$$

it is possible to establish a relation between non-factorizing NNLO mixed corrections and the differences between NLO EW *K*-factors for $V+2$ jet and $V+1$ jet production. To this end, we consider the identity

71 $$\begin{aligned}&\frac{\mathrm{d}}{\mathrm{d}x}\sigma ^{V+2\,\mathrm {jets}}_{\text {NLO} \, {\mathrm {EW}}}(x,{\tau }_{\mathrm {cut}}) = \frac{\mathrm{d}}{\mathrm{d}x}\sigma ^{V+2\,\mathrm {jets}}_{\text {LO} \, {\mathrm {QCD}}}(x,{\tau }_{\mathrm {cut}}) \nonumber \\&\quad \times \left[ \kappa _{\text {NLO} \, {\mathrm {EW}}}^{V+1\,\mathrm {jet}}(x) + \delta \kappa ^{(V)}_{\mathrm {NNLO}\,{\mathrm {mix}}}(x,{\tau }_{\mathrm {cut}})\right] , \end{aligned}$$

which is obtained by multiplying both sides of Eq. (70) by the LO QCD cross section for $pp\rightarrow V+1$ jet and restricting the phase space to real–virtual contributions with $V+2$ jet final states. This restriction is implemented by means of an *N*-jettiness [82] resolution parameter ${\tau }_{\mathrm {cut}}$, as described in more detail below, and the above equation should be understood as the definition of $\delta \kappa ^{(V)}_{\mathrm {NNLO}\,{\mathrm {mix}}}(x,{\tau }_{\mathrm {cut}})$, which will be used as estimator of $\delta \kappa ^{(V)}_{\mathrm {NNLO}\,{\mathrm {mix}}}(x)$ in Eq. (70). In Eq. (71) we use the notation $\kappa _{\text {NLO} \, {\mathrm {EW}}}^{V+1\,\mathrm {jet}}(x)=\kappa _{\text {NLO} \, {\mathrm {EW}}}^{(V)}(x)$, and we keep the $\mu$-dependence as implicitly understood, since the term $\delta \kappa ^{(V)}_{\mathrm {NNLO}\,{\mathrm {mix}}}(x,{\tau }_{\mathrm {cut}})$ is expected to be quite stable with respect to scale variations. Instead, the ${\tau }_{\mathrm {cut}}$ parameter plays an important role since it acts as a cut-off of infrared QCD singularities in the regions where the second jet becomes soft or collinear. Based on the universal behavior of IR QCD effects, such singularities are expected to factorize into identical singular factors on the left- and the right-hand side of Eq. (71). Thus, while the $\delta \kappa ^{(V)}_{\mathrm {NNLO}\,{\mathrm {mix}}}(x,{\tau }_{\mathrm {cut}})$ term on the right-hand side depends on ${\tau }_{\mathrm {cut}}$, this dependence is expected to be free from large ${\tau }_{\mathrm {cut}}$-logarithms and thus reasonably mild.

**Fig. 15:**
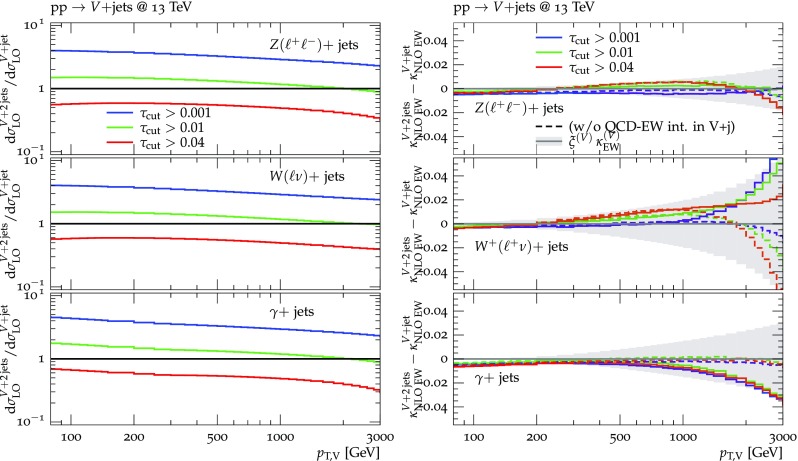
The left plot shows ratios of the different $V+2\,$jets over $V + 1\,$jet predictions at LO for three values of the jettiness resolution parameter $\tau _{\mathrm{cut}}$. The right plot shows the estimator of non-factorizing mixed EW–QCD effects (72), i.e. the difference between the EW *K*-factors for one- and two-jet processes. Results with full EW corrections (solid lines) are compared to the case where QCD–EW bremsstrahlung interference contributions to $pp\rightarrow V+1$ jet are not included (dashed lines). The gray band corresponds to the Ansatz (68) with the $\xi ^{(V)}$ coefficients specified in Eq. (75)

As anticipated above, solving for $\delta \kappa ^{(V)}_{\mathrm {NNLO}\,{\mathrm {mix}}}$ we obtain the relation

72 $$\begin{aligned} \delta \kappa ^{(V)}_{\mathrm {NNLO}\,{\mathrm {mix}}}(x,{\tau }_{\mathrm {cut}})= & {} \kappa ^{V+2\,\mathrm {jets}}_{\text {NLO} \, {\mathrm {EW}}}(x,{\tau }_{\mathrm {cut}}) -\kappa _{\text {NLO} \, {\mathrm {EW}}}^{V+1\,\mathrm {jet}}(x), \end{aligned}$$

which allows us to estimate non-factorizing mixed effects in terms of the difference between the $V+2$-jet and $V+1$-jet EW $\kappa$-factors. To this end, we will match the estimator (72) to the Ansatz (68). More precisely, we will fix the free coefficients $\xi ^{(V)}$ in Eq. (68) in such a way that

73 $$\begin{aligned} \xi ^{(V)}\, \kappa _{\text {NLO} \,{\mathrm {EW}}}^{V+1\,\mathrm {jet}}(x)\gtrsim & {} \kappa ^{V+2\,\mathrm {jets}}_{\text {NLO} \, {\mathrm {EW}}}(x,{\tau }_{\mathrm {cut}}) -\kappa _{\text {NLO} \, {\mathrm {EW}}}^{V+1\,\mathrm {jet}}(x) \end{aligned}$$

for the whole *x*-spectrum and within an appropriately chosen ${\tau }_{\mathrm {cut}}$ range. Thanks to the cancellation of IR QCD singularities in Eq. (72), the resulting $\xi ^{(V)}$ coefficients should be reasonably stable with respect to the choice of the resolution parameter. Thus, ${\tau }_{\mathrm {cut}}$ can be varied in a rather wide range. In principle one could even consider the ${\tau }_{\mathrm {cut}}\rightarrow 0$ limit of Eq. (73). However, given that two-loop mixed EW–QCD contributions are not taken into account, this limit does not converge towards the full NNLO result corresponding to ${\tau }_{\mathrm {cut}}=0$. Moreover, for very small values of ${\tau }_{\mathrm {cut}}$ the numerator and denominator of $\kappa ^{V+2\,\mathrm {jets}}_{\text {NLO} \, {\mathrm {EW}}}(x,{\tau }_{\mathrm {cut}})$ are dominated by universal ${\tau }_{\mathrm {cut}}$-logarithms that should cancel against virtual two-loop terms, and since such logarithms factorize, their dominance can result in an underestimation of non-factorizing effects. Vice versa, excessively large values of ${\tau }_{\mathrm {cut}}$ can lead to an overestimation of non-factorizing effects. This is due to the fact that increasing ${\tau }_{\mathrm {cut}}$ enhances the difference between EW $\kappa$-factors in Eq. (73) but also suppresses the cross section of the $V+2$-jet subprocess, rendering it a less and less significant estimator of the behavior of mixed corrections for inclusive $V+$ jet production. Thus, excessively small or large values of ${\tau }_{\mathrm {cut}}$ should be avoided.

Based on the above considerations, for the fit of the $\xi ^{(V)}$ coefficients we require that Eq. (73) is fulfilled in a wide ${\tau }_{\mathrm {cut}}$-range while keeping the $\sigma ^{V+2\,\mathrm {jet}}/\sigma ^{V+1\,\mathrm {jet}}$ ratio at order one, in such a way that the $V+2\,$jet cross section is neither too suppressed nor too enhanced. This procedure is implemented using an *N*-jettiness cut parameter [82]. More precisely, we use the dimensionless one-jettiness parameter

74 $$\begin{aligned} {\tau }_1 = \sum _k \text {min}_i \left\{ \frac{2 p_i \cdot q_k}{Q_i\,\sqrt{\hat{s}}}\right\} , \end{aligned}$$

where the $p_i$ are light-like vectors for each of the initial beams and the hardest final-state jet, and the $Q_i$ characterize their respective hardness, which we set as $Q_i = 2 E_i$. The hardest final-state jet is defined by applying an anti-$k_\mathrm{{T}}$ algorithm with R=1 to all final-state partons.^15^ The $q_k$ denote the four-momenta of any such final-state parton, and $\sqrt{\hat{s}}$ is the partonic center-of-mass energy. All quantities are defined in the hadronic center-of-mass system.

To isolate two-jet configurations against one-jet configurations we require $\tau _1>{\tau }_{\mathrm {cut}}$, and the cut is varied in the range $0.001 \le {\tau }_{\mathrm {cut}}\le 0.04$. As demonstrated in Fig. 15, this choice keeps the $\sigma ^{V+2\,\mathrm {jet}}/\sigma ^{V+1\,\mathrm {jet}}$ ratio around order one, as desired. Moreover, we observe that the estimator (73) remains quite stable with respect to ${\tau }_{\mathrm {cut}}$ variations (see the solid lines in the right plot). Non-factorizing effects turn out to be generally very small. They exceed the percent level only in the TeV tails of the distributions. As illustrated by the gray band in Fig. 15 (right), setting

75 $$\begin{aligned} \xi ^{Z}=0.1, \quad \xi ^{W}=0.2, \quad \xi ^{\gamma }=0.4, \end{aligned}$$

guarantees an acceptable matching of the Ansatz (68) to the estimator (73). More precisely, for $W+$ jet production the shape of the Ansatz (68) tends to overestimate the uncertainty in the $p_{\mathrm {T}}$ range between one and two TeV. However, we have checked that the Ansatz becomes much less adequate if the full EW correction in Eq. (67) is replaced by its non-Sudakov part.

The rather small values of the $\xi ^{(V)}$ coefficients confirm that the bulk of the EW and QCD corrections factorize. However, in the case of $W+$ jet and $\gamma +$ jet production, the relative size of non-factorizing corrections appears to be rather significant. This is due to the behavior of the EW $\kappa$-factors in the multi-TeV region, where the difference between the EW $\kappa$-factors for $pp\rightarrow V+1$ jet and $pp\rightarrow V+2$ jet is enhanced by the presence of mixed EW–QCD interference contributions in channels of type $qq\rightarrow qq V$ (see the contributions of type a.5 in Sect. 4.2). More precisely, EW–QCD interference effects of $\mathcal {O}(\alpha _{\mathrm {S}}\alpha ^2)$ enhance the EW corrections to $pp\rightarrow V+1$ jet as a result of the opening of the *qq* channel at NLO EW, while in $pp\rightarrow V+2$ jet the EW *K*-factor is not enhanced since the *qq* channel is already open at LO. Based on this observation, and also on the fact that the main effect of the opening of the *qq* channel is already reflected in the NLO QCD *K*-factor for $V+1\,$jet production, the above-mentioned EW–QCD interference effects could be excluded from the factorization prescription (64) and treated as a separate contribution. As illustrated by the dashed curves in Fig. 15, this approach would lead to a drastic reduction of non-factorizing effects, especially for $\gamma +$ jet production. Nevertheless, given that the effects observed in Fig. 15 are subdominant with respect to current PDF and statistical uncertainties, in the present study we refrain from implementing such a splitting.

### Combination of QCD and EW corrections with related uncertainties

Based on the above analysis, we recommend to combine QCD and EW corrections according to the multiplicative prescription (67), treating the non-factorizing term (68) as uncertainty and using the estimated $\xi ^{(V)}$ factors given in Eq. (75). Including QCD and EW uncertainties as specified in Eq. (39) and Eq. (58), this leads to the combination formula

76 $$\begin{aligned}&K^{(V)}_{\mathrm {TH}}(x,{\varvec{\varepsilon }}_{\mathrm {QCD}},{\mathbf {\varepsilon }}_{\mathrm {EW}},\varepsilon _{\mathrm {mix}}) \nonumber \\&\quad = K^{(V)}_{\mathrm {TH},\otimes }(x,{\varvec{\varepsilon }}_{\mathrm {QCD}},{\mathbf {\varepsilon }}_{\mathrm {EW}}) + \varepsilon _{\mathrm {mix}}\, \delta K^{(V)}_{{\mathrm {mix}}}(x)\nonumber \\&\quad = \left[ K_{\mathrm {N}^k\mathrm {LO}}^{(V)}(x) +\sum _{i=1}^3\varepsilon _{{\mathrm {QCD}},i}\,\delta ^{(i)} K^{(V)}_{\mathrm {N}^k\mathrm {LO}}(x) \right. \nonumber \\&\qquad \left. + \sum _{i=1}^{107}\varepsilon _{\mathrm {PDF},i}\,\delta ^{(i)} K^{(V)}_{\mathrm {PDF}}(x) \right] \nonumber \\&\qquad \times \left[ 1 + \kappa _{{\mathrm {EW}}}^{(V)}(x) +\sum _{i=1}^3\varepsilon ^{(V)}_{{\mathrm {EW}},i}\,\delta ^{(i)}\kappa ^{(V)}_{\mathrm {EW}}(x) \right] \nonumber \\&\qquad +\, \varepsilon _{\mathrm {mix}}\, \delta K^{(V)}_{{\mathrm {mix}}}(x), \end{aligned}$$

where the uncertainty associated with non-factorizing mixed EW–QCD terms reads

77 $$\begin{aligned} \delta K^{(V)}_{{\mathrm {mix}}}(x)= & {} \xi ^{(V)} \left[ K_{\mathrm {N}^k\mathrm {LO}}^{(V)}(x)-1\right] \kappa _{{\mathrm {EW}}}^{(V)}(x)\nonumber \\= & {} \xi ^{(V)} \left[ K^{(V)}_{\mathrm {TH},\oplus }(x)- K^{(V)}_{\mathrm {TH},\otimes }(x) \right] . \end{aligned}$$

The related nuisance parameter, $\varepsilon _{\mathrm {mix}}$, should be Gaussian distributed with one standard deviation corresponding to the range $\varepsilon _{\mathrm {mix}}\in [-1,+1]$. Given that mixed uncertainties have been estimated using a proxy of the full NNLO QCD–EW calculation, it would be reasonable to assume some degree of correlation across different $V+$ jet processes. However, for simplicity in this study we keep $\varepsilon _{\mathrm {mix}}$ variations fully uncorrelated, bearing in mind that this approach is probably too conservative.

**Fig. 16:**
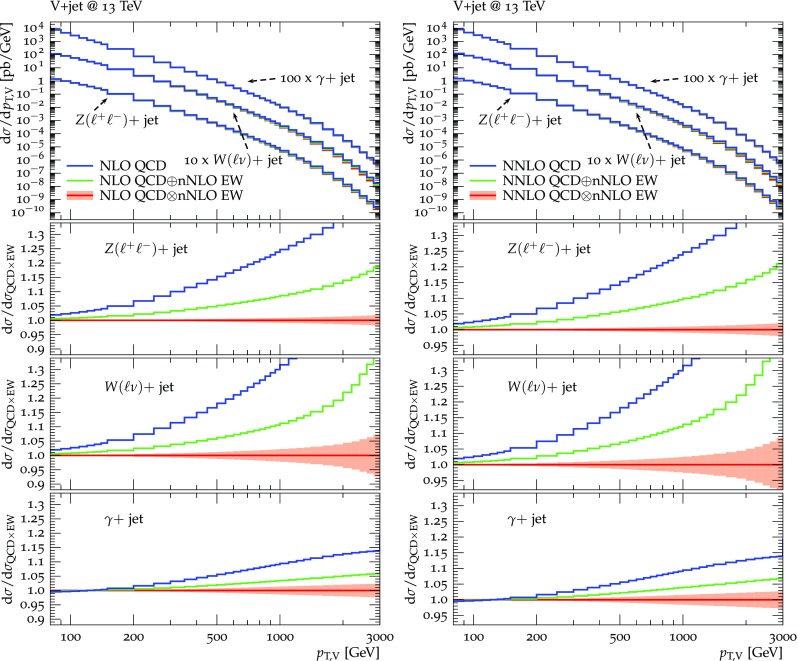
Comparison of additive (green) and multiplicative (red) combination of (N)NLO QCD and nNLO EW corrections for various $pp\rightarrow V+$jet processes at 13 TeV. The red band corresponds to the mixed QCD–EW uncertainty (77). The (N)NLO QCD result without EW corrections is shown in blue. The combination at NLO QCD is shown on the left and at NNLO QCD on the right

In Fig. 16 we compare the additive and multiplicative combinations of QCD and EW corrections showing also the corresponding uncertainty estimate (77) for various $V+$jet processes.

## Summary and conclusions

The precise control of SM backgrounds, and notably of $pp \rightarrow Z(\nu {\bar{\nu }})+$ jets, is crucial in order to maximize the potential of MET+jets searches at the LHC. Such backgrounds can be predicted directly using QCD and EW calculations. Alternatively, QCD and EW calculations can be used to relate them to experimental data for similar $V+$ jet production processes, i.e. $pp\rightarrow \gamma +$ jets, $pp \rightarrow W(\ell \nu )+$ jets and $pp \rightarrow Z(\ell ^+\ell ^-)+$ jets.

In this article we have presented predictions for inclusive vector-boson $p_\mathrm {T}$ distributions based on the most advanced calculations available today, bringing together results from a number of groups so as to have perturbative QCD to NNLO accuracy, EW corrections to NLO accuracy and additionally the inclusion of two-loop EW Sudakov logarithms.

A substantial part of our study concerned uncertainty estimates. In particular we proposed and applied various new approaches for uncertainty estimates and correlations across processes and $p_\mathrm {T}$ regions.

We defined the uncertainties due to normal QCD scale variations in a way that gives a strong correlation across different $p_\mathrm {T}$ regions, Eq. (33). We then supplemented it with a shape uncertainty that is anti-correlated across $p_\mathrm {T}$, Eqs. (35)–(36). To address the long-standing problem of evaluating the correlations between uncertainties for different processes, we separated the uncertainty into process-independent and process-dependent components. The universal component was taken to be composed of the overall scale and shape uncertainties for the reference $Z+\mathrm {jet}$ process. The process-dependent component, which is generally small, was determined by considering the difference between suitably normalized *K*-factors for the different processes, Eq. (38). This amounts to a conservative choice of taking the uncertainty on ratios as the difference between the best available prediction and the one at one order lower.

Special attention was devoted to the correlation of $Z/W+$ jet and $\gamma +$ jet production. In that case a substantial non-universal contribution is associated with the masslessness of the photon and the need to control collinear divergent $q\rightarrow q\gamma$ radiation through a photon-isolation prescription. We introduced a novel photon-isolation prescription with a dynamically chosen isolation radius, Eq. (11), designed to suppress $q\rightarrow \gamma q$ radiative effects in a way that is similar to the effect of the masses of the *Z* and *W* bosons in the case of $q\rightarrow V q$ splittings at large $p_\mathrm {T}$. Such a dynamic isolation allows one to split $\gamma +$jet production into a quasi-universal part, which can be treated on the same footing as $Z+\mathrm {jet}$ and $W+\mathrm {jet}$ production, and a non-universal part which is kept uncorrelated. The non-universal part is given by the difference between the cross sections with conventional and dynamic photon-isolation prescriptions.

For pure EW corrections we considered three uncertainty sources for unknown higher-order contributions. These address unknown Sudakov logarithms beyond NNLO and/or NLL accuracy, as well as unknown hard (non-Sudakov) EW corrections beyond NLO and process-correlation effects.

One potentially large source of uncertainty arises from mixed QCD and EW corrections, given that both $\mathcal {O}(\alpha _{\mathrm {S}})$ and $\mathcal {O}(\alpha )$ NLO corrections can be large and that the $\mathcal {O}(\alpha \alpha _{\mathrm {S}})$ NNLO corrections are not currently known. We chose a multiplicative scheme for combining EW and QCD corrections. To obtain an estimate of unknown $\mathcal {O}(\alpha \alpha _{\mathrm {S}})$ corrections not captured by this factorized Ansatz, we studied the NLO EW corrections to $V+2$ jet production, which represent the real–virtual part of a full $\mathcal {O}(\alpha \alpha _{\mathrm {S}})$ calculation for $V+$ jet production. Based on this analysis, we concluded that it is reasonable to assume that the multiplicative combination of QCD and EW corrections describes the full $\mathcal {O}(\alpha \alpha _{\mathrm {S}})$ correction with a relative uncertainty that varies between 10 and 20% for $pp\rightarrow W/Z+$ jet and 40% for $pp\rightarrow \gamma +$ jet.

Overall, QCD corrections are substantial, a few tens of percent at NLO, and up to $10\%$ at NNLO. The NNLO results are consistent with the NLO predictions within our prescription for the uncertainty bands of the latter. This is true not just for absolute cross sections and their shapes, but also for ratios of cross sections. These ratios are remarkably stable across LO, NLO and NNLO QCD corrections; see Fig. 8. Using dynamic photon isolation, this statement holds true also for the $\gamma +\mathrm {jet}$ process at $p_\mathrm {T}\gtrsim 300~\text {GeV}$.

The EW corrections to $V+$jet cross sections amount to a few tens of percent in the TeV region; see Fig. 9. In the ratios they cancel only in part, due to the sensitivity of EW effects to the SU(2) charges of the produced vector bosons. At the TeV scale, the NNLO Sudakov logarithms can reach the several percent level and their systematic inclusion is an important ingredient in order to achieve percent precision at very high $p_\mathrm {T}$.

**Fig. 17:**
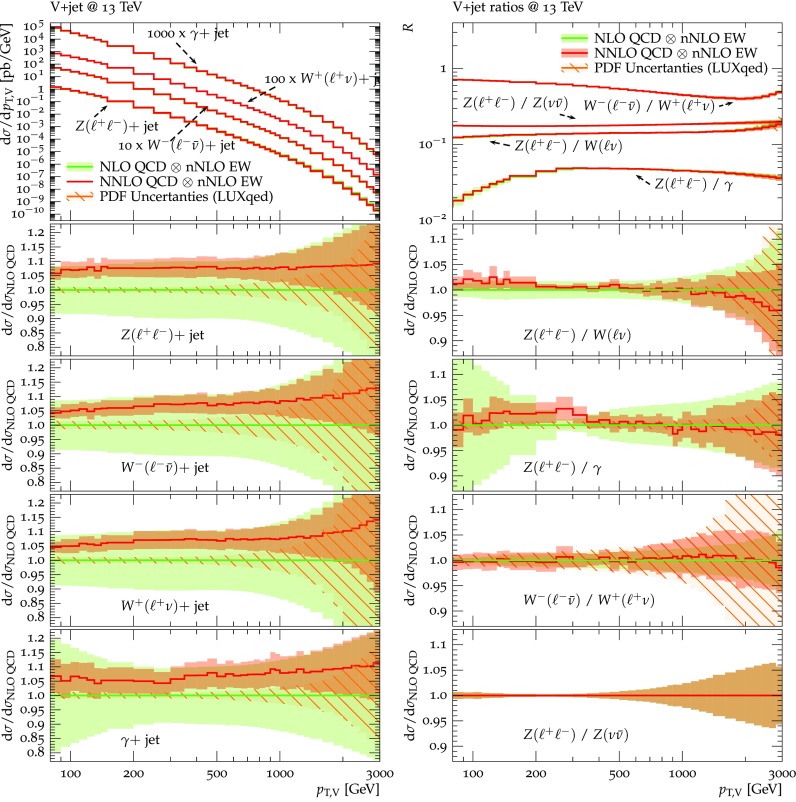
Predictions at NLO QCD $\otimes$ nNLO EW and NNLO QCD $\otimes$ nNLO EW for $V+$ jet spectra (left) and ratios (right) at 13 TeV. The lower frames show the relative impact of NNLO corrections and theory uncertainties normalized to NLO QCD $\otimes$ nNLO EW. The green and red bands correspond to the combination (in quadrature) of the perturbative QCD, EW and mixed QCD-EW uncertainties, according to Eq. (76) at NLO QCD $\otimes$ nNLO EW and NNLO QCD $\otimes$ nNLO EW respectively. PDF uncertainties based on LUXqed_plus_PDF4LHC15_nnlo are shown at NLO QCD as separate hashed orange bands

**Fig. 18:**
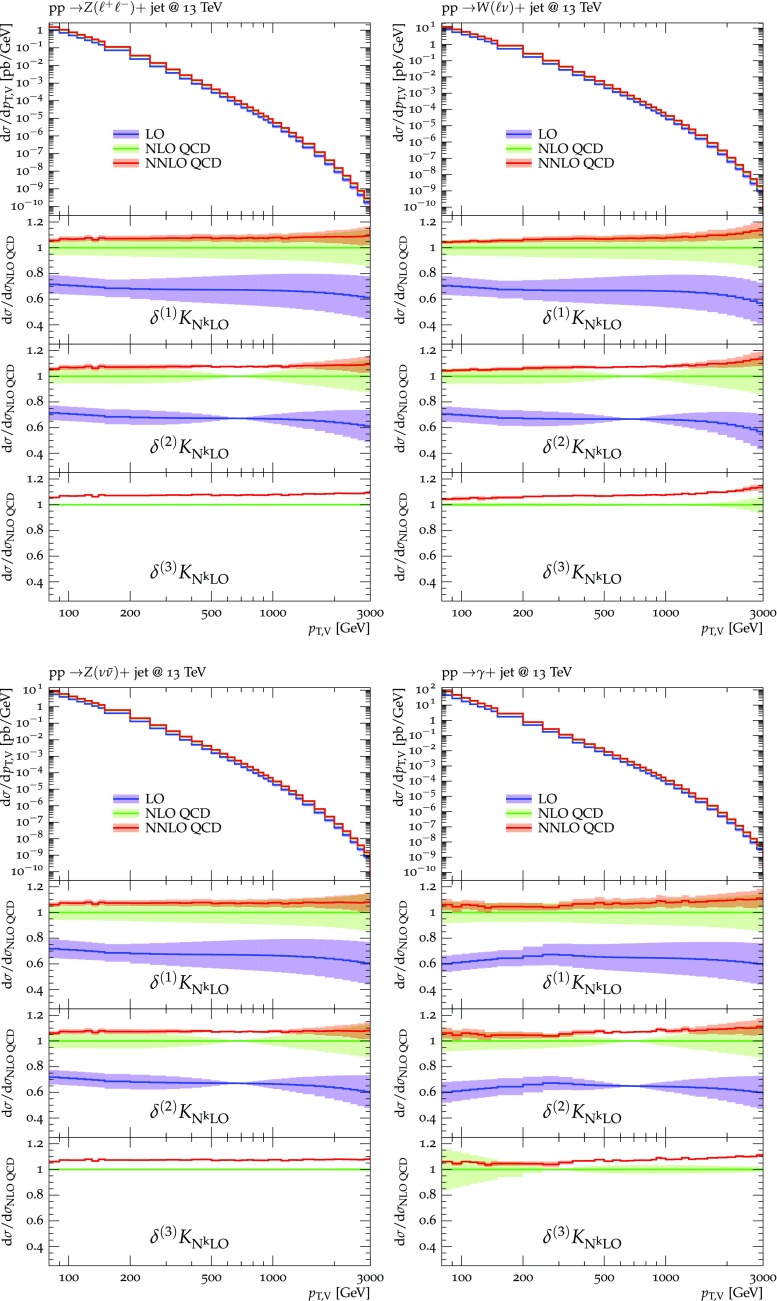
Higher-order QCD predictions and uncertainties for various $pp\rightarrow V+$jet processes at 13 TeV. Absolute predictions at LO and NLO QCD are displayed in the main frame. In the ratio plots all results are normalized to NLO QCD, and the bands correspond to the three types of QCD uncertainties, $\delta ^{(i)}K_{\mathrm {N}^k\mathrm {LO}}$, i.e. scale uncertainties (33), shape uncertainties (35) and process-correlation uncertainties (38)

**Fig. 19:**
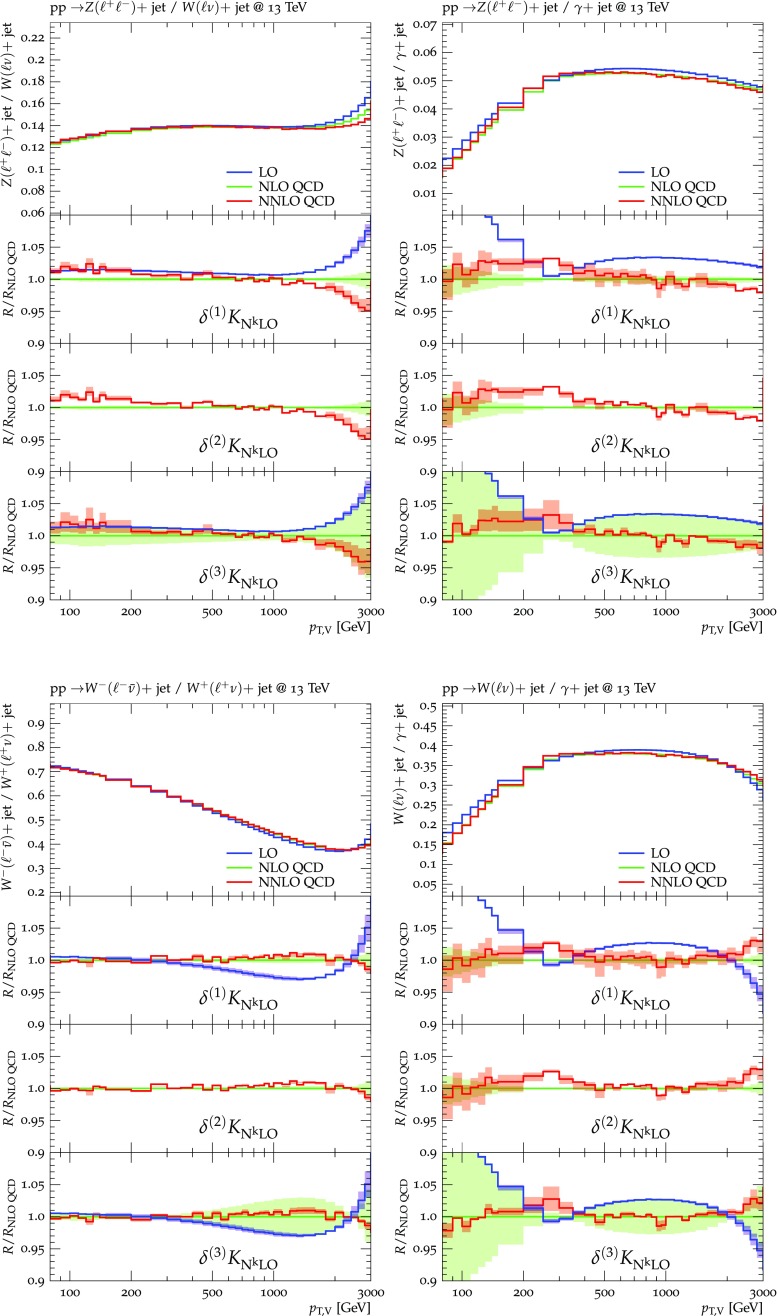
Ratios of $p_\mathrm {T}$-distributions for various $pp\rightarrow V+$jet processes at LO and NLO QCD. The related scale uncertainties (33), shape uncertainties (35) and process-correlation uncertainties (38) are correlated amongst all processes as discussed in Sect. 4.1

**Fig. 20:**
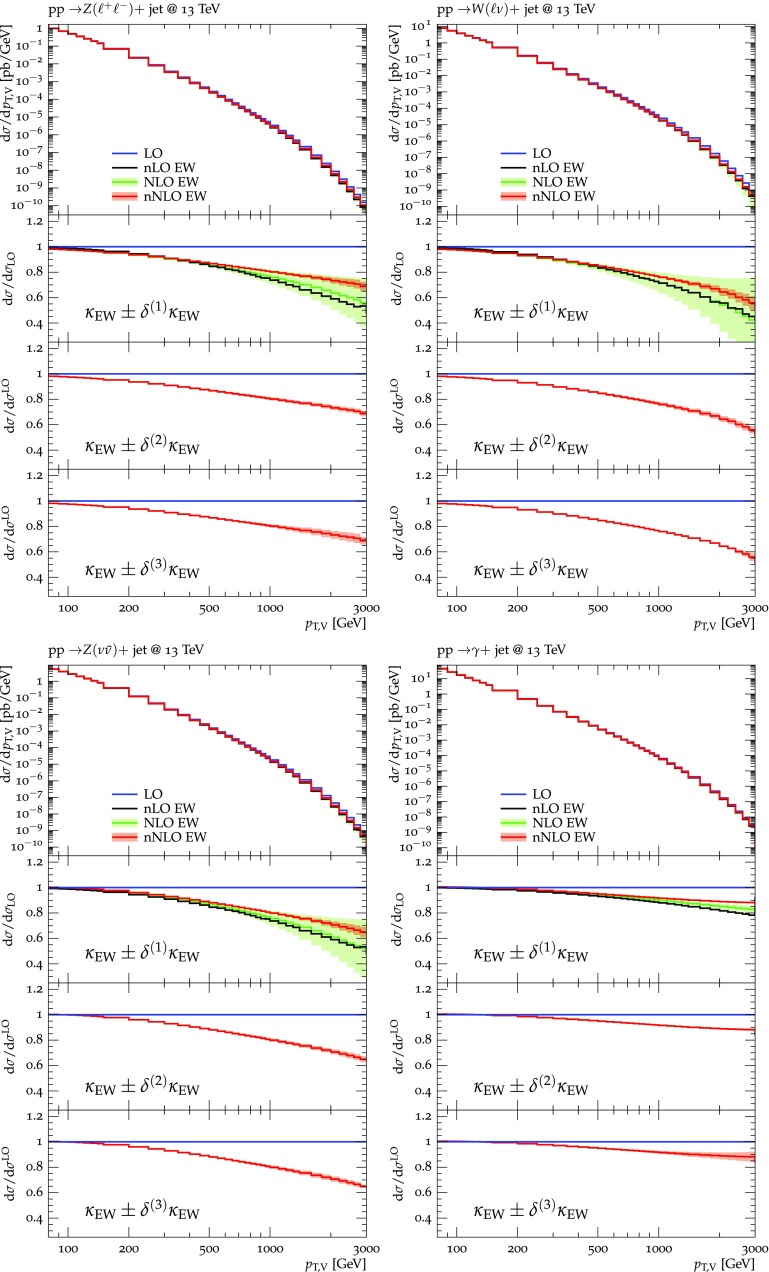
Higher-order EW predictions and uncertainties for different $pp\rightarrow V+$ jet processes at 13 TeV. The main frames display absolute predictions at LO (blue), NLO EW (green) and nNLO EW (red), as well as NLL Sudakov logarithms at NLO (black). The latter are dubbed nLO EW. In the ratio plots all results are normalized to LO. The bands correspond to the three types of EW uncertainties, $\delta ^{(i)}\kappa ^{(V)}_{{\mathrm {EW}}}$. At nNLO EW (red bands) they are defined in Eqs. (52), (53) and (56), while at NLO EW (green band) only the uncertainty $\delta ^{(1)}\kappa ^{(V)}_{\text {NLO} \,{\mathrm {EW}}}$, defined in Eq. (57), is plotted

**Fig. 21:**
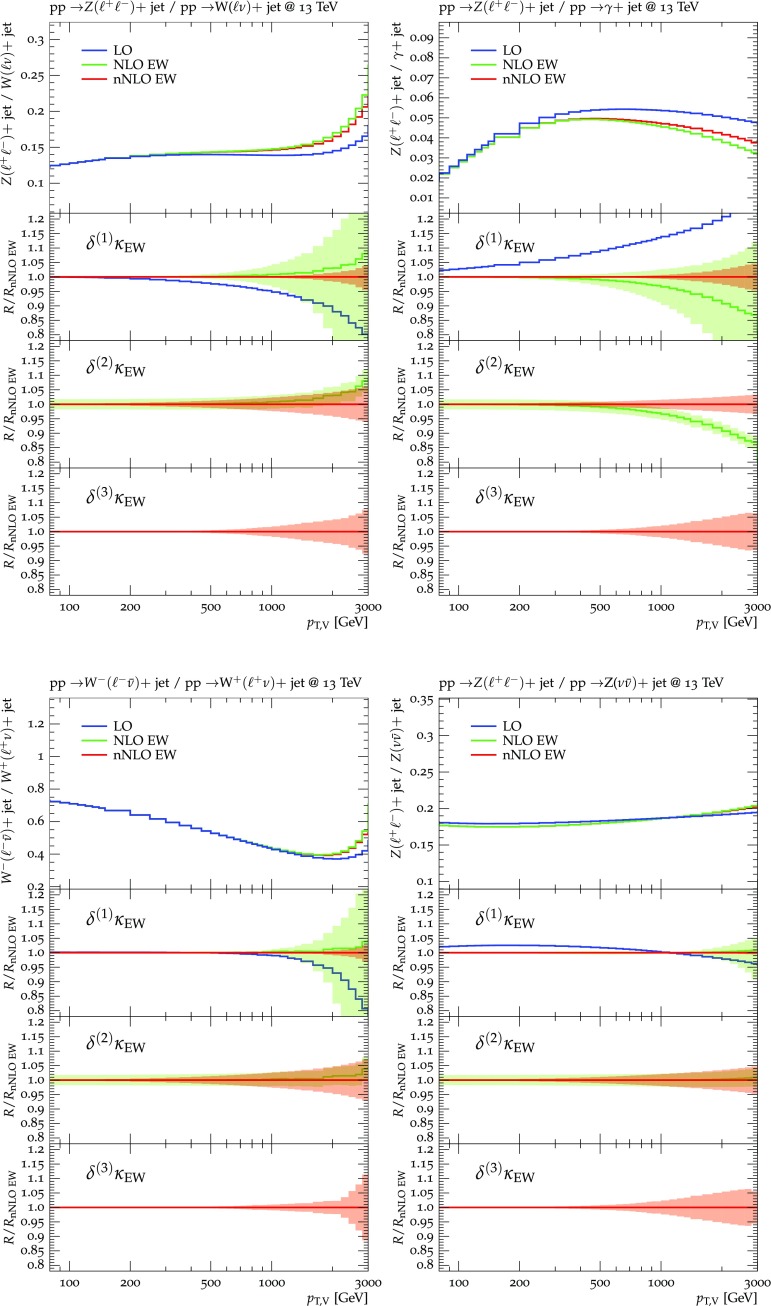
Ratios of $p_\mathrm {T}$-distributions for various $pp\rightarrow V+$jet processes at LO, NLO EW and nNLO EW. The related EW uncertainties, $\delta ^{(i)}\kappa ^{(V)}_{{\mathrm {EW}}}$, are defined in Eqs. (52), (53) and (56) at nNLO and in Eq. (57) at NLO. The uncertainty $\delta ^{(1)}\kappa ^{(V)}_{{\mathrm {EW}}}$ is correlated amongst processes, while $\delta ^{(2)}\kappa ^{(V)}_{{\mathrm {EW}}}$ and $\delta ^{(3)}\kappa ^{(V)}_{{\mathrm {EW}}}$ are uncorrelated

In Fig. 17 we summarize our uncertainty estimates for the different $V+$jet processes and process ratios. Here we combine in quadrature all sources of perturbative uncertainties at N(N)LO QCD $\otimes$ nNLO EW and we overlay the remaining PDF uncertainties. For convenience, PDF variations have been assessed using NNLO PDFs in combination with NLO QCD calculations, but they can be safely applied to the NNLO QCD results. The nominal $p_{\mathrm {T}}$ distributions at N(N)LO QCD $\otimes$ nNLO EW are constrained at the 10(5)% level up to about 1 TeV and at the 20(10)% level up to about 2 TeV. In the process ratios these uncertainties cancel to a large extent. In particular, in the *Z* / *W* ratio remaining uncertainties are at the level of only 1–2% up to 1 TeV and below 5% up to 2 TeV. Similarly, the $Z/\gamma$ ratio is constrained at the 5% level up to 2 TeV. Noteworthy, including the NNLO QCD corrections the process ratios remain very stable and in particular within the uncertainty estimates based on NLO QCD. This reflects the fact that QCD uncertainties are very well under control: taking at face value the NNLO QCD systematics we are at the level of a few percent all the way up to the multi-TeV scale (see Fig. 8), and at large $p_{\mathrm {T}}$ we are dominated by EW and PDF uncertainties. The latter are below the perturbative uncertainties in all nominal distributions and all but the $W^-/W^+$ ratio, where a precise measurement at high $p_{\mathrm {T}}$ could help to improve PDF fits. In this respect, we note that the theoretical uncertainty for the $W^-/W^+$ ratio is entirely dominated by mixed QCD–EW effects and is most likely overestimated due to our conservative assumption of keeping such uncertainties uncorrelated across processes (see Sect. 4.6).

We also discussed photon-induced contributions and QED corrections to PDFs. In this context, for a precise prediction of the $\gamma$-PDF we have advocated the use of the LUXqed_plus_PDF4LHC15_nnlo PDFs, which implement a data-driven determination of the $\gamma$-PDF. For a consistency treatment of $\mathcal {O}(\alpha )$ effects in the PDFs, the LUXqed_plus_PDF4LHC15_nnlo distributions should be used in all photon-, quark-, and gluon-induced channels.^16^ Photon-induced effects are negligible in $Z+$ jet and $\gamma +$ jet production, but their impact on $pp\rightarrow W+$ jet, and thus on the *W* / *Z* and $W/\gamma$ ratios, can reach the 5% level at the TeV scale^17^ (see Fig. 12).

Our predictions are provided in the form of tables for the central predictions and for the different uncertainty sources. Each uncertainty source is to be treated as a 1-standard deviation uncertainty and pragmatically associated with a Gaussian-distributed nuisance parameter.

The predictions are given at parton level as distributions of the vector boson $p_\mathrm {T}$, with loose cuts and inclusively over other radiation. They are intended to be propagated to an experimental analysis using Monte Carlo parton shower samples whose inclusive vector-boson $p_\mathrm {T}$ distribution has been reweighted to agree with our parton-level predictions. The impact of additional cuts, non-perturbative effects on lepton isolation, etc., can then be deduced from the Monte Carlo samples. The additional uncertainties associated with the Monte Carlo simulation are expected to be relatively small, insofar as the vector-boson $p_\mathrm {T}$ distribution that we calculate is closely connected to the main experimental observables used in MET$+$jets searches.

Some caution is needed in implementing the results of this paper: for example the uncertainty prescriptions are tied to the use of the central values that we provide. If an experiment relies on central values that differ, e.g. through the use of MC samples that are not reweighted to our nominal predictions, then the uncertainty scheme that we provide may no longer be directly applicable. Furthermore, for searches that rely on features of the event other than missing transverse momentum, one should be aware that our approach might need to be extended. This would be the case notably for any observable that relies directly on jet observables, whether related to the recoiling jet or vetoes on additional jets.

Overall, it is possible to obtain precise theoretical control both for vector-boson $p_\mathrm {T}$ distributions, and for their ratios, at the level of a few percent. We expect this precision, across a wide range of $p_\mathrm {T}$, to be of significant benefit in MET+jets searches, notably enabling reliable identification or exclusion of substantially smaller BSM signals than was possible so far. In fact, since the release of the first version of this paper, the background estimates we propose here have been adopted in analyses by ATLAS [83] and CMS [84].

## Appendix A: Theoretical predictions and uncertainties

Predictions for the various $pp\rightarrow V+$ jet processes listed in Table 2 with $\sqrt{s}=13~\text {TeV}$ are provided at http://lpcc.web.cern.ch/content/dark-matter-wg-documents.

The various predictions and related uncertainties at the highest available perturbative order, i.e. NNLO QCD and nNLO EW, as well as the labels of the corresponding histograms are listed in Table 3. Predictions with uncertainties at NLO and additional building blocks for the construction of the uncertainties at the various perturbative orders can also be found in Table 3.

**Table 2:** List of processes, highest available QCD and EW order, and process labels used in data files (see Table 3). Predictions for $pp\rightarrow \nu _\ell {\bar{\nu }}_\ell +$ jet are available only at NLO QCD, but corresponding NNLO QCD corrections and uncertainties can be taken from $pp\rightarrow \ell ^+\ell ^-+$ jet

Process	QCD order	EW order	Label
$pp\rightarrow \ell ^+\nu _\ell /\ell ^-{\bar{\nu }}_\ell +$ jet	NNLO QCD	nNLO EW	evj
$pp\rightarrow \nu _\ell {\bar{\nu }}_\ell +$ jet	NLO QCD	nNLO EW	vvj
$pp\rightarrow \ell ^+\ell ^-+$ jet	NNLO QCD	nNLO EW	eej
$pp\rightarrow \gamma +$ jet	NNLO QCD	nNLO EW	aj

All ingredients and related uncertainties should be combined as indicated in Eqs. (62) and (76), and we recall that all nuisance parameters in Eq. (76) should be Gaussian distributed with one standard deviation corresponding to the range $[-1,+1]$ for all $\varepsilon _{{\mathrm {QCD}},i}$, $\varepsilon _{\mathrm {PDF},i}$, $\varepsilon _{{\mathrm {EW}},i}$ and $\varepsilon _{\mathrm {mix}}$. In the implementation of the various relative uncertainties, i.e. $\delta ^{(i)} K_{\mathrm {N}^k\mathrm {LO}}$, $\delta ^{(i)} K_{\mathrm {PDF}}$, and $\delta ^{(i)} K_{{\mathrm {EW}}}$, it is crucial to take into account their correct normalization according to Eqs. (62) and (76). For instance, at $\mathrm {NNLO}\,{\mathrm {QCD}}\otimes \mathrm {nNLO}\,{\mathrm {EW}}$ the relative impact of QCD and EW uncertainties should be $\delta ^{(i)} K_{\mathrm {NNLO}}/K_{\mathrm {NNLO}}$ and $\delta ^{(i)} K_{{\mathrm {EW}}}/(1+\kappa _{\mathrm {EW}})$, respectively.

Concerning QCD contributions, predictions at NNLO QCD should be combined with uncertainties at the same order. However, before higher-order QCD calculations are thoroughly validated against high-statistics measurements at moderate transverse momenta, theory uncertainties should be assessed in a more conservative way. To this end, we advocate the usage of NNLO QCD nominal predictions in combination with NLO QCD uncertainties, while keeping all EW effects at nNLO EW level.

All predictions and uncertainties for $pp\rightarrow \gamma +$ jet are based on the dynamic photon-isolation prescription introduced in Sect. 3.1. As explained therein, this requires an extra $\gamma +$ jet specific uncertainty, which needs to be evaluated by means of a separate reweighting in a standard Frixione isolation setup with fixed cone. Corresponding theoretical predictions at NLO QCD are denoted as $K^{(\gamma ,\mathrm {fix})}_{\text {NLO}}(x)$ in Table 3.

**Table 3:** Naming scheme for the theoretical predictions and uncertainties described in Sect. 4. The upper part lists the highest available perturbation order, while the predictions in the lower part are included for completeness. The last column indicates the correlation of the uncertainties across different $V+$jets processes. The actual distribution names are x=pTV and the individual processes are available in the files proc.dat with process names proc=eej,vvj,evj,aj, as defined in Table 2. Absolute predictions for $p_\mathrm {T}$ distributions are in pb/GeV

Prediction	Equations	Label	Correlation
$\frac{\mathrm{d}}{\mathrm{d}x}\sigma ^{(V)}_{\text {LO} \,{\mathrm {QCD}}}({\varvec{\mu }}_0)$ [pb/GeV]	(39)	proc_x_LO	–
$K^{(V)}_{\mathrm {NNLO}}(x)$	(39), (32)	proc_x_K_NNLO	–
$\delta ^{(1)}K^{(V)}_{\mathrm {NNLO}}(x)$	(39), (33)	proc_x_d1K_NNLO	Yes
$\delta ^{(2)}K^{(V)}_{\mathrm {NNLO}}(x)$	(39), (35)	proc_x_d2K_NNLO	Yes
$\delta ^{(3)}K^{(V)}_{\mathrm {NNLO}}(x)$	(39), (38)	proc_x_d3K_NNLO	Yes
$K^{(\gamma ,\mathrm {dyn})}_{\text {NLO}}(x)$	(39), (32), (13)	aj_x_K_NLO	–
$K^{(\gamma ,\mathrm {fix})}_{\text {NLO}}(x)$	(39), (32), (14)	aj_x_K_NLO_fix	–
$\kappa ^{(V)}_{{\mathrm {EW}}}(x)=\kappa ^{(V)}_{\mathrm {nNLO}\,{\mathrm {EW}}}(x)$	(40)–(43), (58)	proc_x_kappa_EW	–
$\delta ^{(1)}\kappa ^{(V)}_{\mathrm {nNLO}\,{\mathrm {EW}}}(x)$	(52), (58)	proc_x_d1kappa_EW	Yes
$\delta ^{(2)}\kappa ^{(V)}_{\mathrm {nNLO}\,{\mathrm {EW}}}(x)$	(53), (58)	proc_x_d2kappa_EW	No
$\delta ^{(3)}\kappa ^{(V)}_{\mathrm {nNLO}\,{\mathrm {EW}}}(x)$	(56), (58)	proc_x_d3kappa_EW	No
$\delta K^{(V)}_{{\mathrm {mix}}}(x)$	(75), (77)	proc_x_dK_NLO_mix	Yes
$\frac{\mathrm{d}}{\mathrm{d}x}\sigma ^{(V)}_{\text {LO} \,\gamma -\mathrm{ind.}}$ [pb/GeV]	(25)	proc_x_gammaind_LO	–
$\delta ^{(i)}K^{(V)}_{\mathrm {PDF}}(x)$	(61)	proc_x_dK_PDF_i	Yes
$K^{(V)}_{\text {LO}}(x)$	(39)	proc_x_K_LO	–
$K^{(V)}_{\text {NLO}}(x)$	(39), (32)	proc_x_K_NLO	–
$\delta ^{(1)}K^{(V)}_{\text {NLO}}(x)$	(39), (33)	proc_x_d1K_NLO	Yes
$\delta ^{(2)}K^{(V)}_{\text {NLO}}(x)$	(39), (35)	proc_x_d2K_NLO	Yes
$\delta ^{(3)}K^{(V)}_{\text {NLO}}(x)$	(39), (38)	proc_x_d3K_NLO	Yes
$\delta ^{(1)}K^{(V)}_{\text {LO}}(x)$	(39), (33)	proc_x_d1K_LO	Yes
$\delta ^{(2)}K^{(V)}_{\text {LO}}(x)$	(39), (35)	proc_x_d2K_LO	Yes
$\kappa ^{(V)}_{\text {NLO} \,{\mathrm {EW}}}(x)$	(40)–(43), (58)	proc_x_kappa_NLO_EW	–
$\kappa ^{(V)}_{\mathrm {NNLO}\,\mathrm {Sud}}(x)$	(40)–(43), (58)	proc_x_kappa_NNLO_Sud	–

## Appendix B: QCD and EW uncertainties

In this appendix we present a series of technical plots that illustrate the relative importance of the various sources of QCD and EW uncertainties discussed in Sects. 4.1–4.2. The impact of individual QCD uncertainties, $\delta ^{(i)}K_{\mathrm {N}^k\mathrm {LO}}$, in $p_\mathrm {T}$ spectra and ratios is illustrated in Figs. 18 and 19. Similar plots for the three types of EW uncertainties, $\delta ^{(i)}\kappa ^{(V)}_{{\mathrm {EW}}}$, are shown in Figs. 20 and 21.

## Footnotes

1 For a recent comprehensive review of DM models leading to this class of signatures; see e.g. [4].

2 Other backgrounds (such as QCD multijets, $t\bar{t}$ or pairs of gauge bosons) are suppressed, and their contribution to the overall uncertainty is well below the percent level.

3 This procedure should be restricted to variables $x'$ that can be described with good accuracy both in perturbative calculations and in the MC simulations.

4 The same isolation prescription used for theory predictions should be applied also to their MC counterparts $\mathrm {d}\sigma _\mathrm{MC}/$d*x* in the context of the reweighting procedure.

5 Diboson backgrounds, including $pp\rightarrow V\gamma$, can be included through separate Monte Carlo samples in the experimental analyses.

6 See e.g. the comparison of NNLOPS against fixed-order predictions in Fig. 3 of Ref. [29].

7 Besides loop diagrams with top quarks and Higgs bosons, the NLO EW corrections to $pp\rightarrow W^\pm +$ jet receive $\mathcal {O}(\alpha ^2\alpha _{\mathrm {S}})$ bremsstrahlung contributions from $qb\rightarrow q' W^\pm b$ channels that involve *s*-channel top-quark propagators and thus require a finite top-quark width, for which we use the NLO QCD value $\Gamma _t=1.339~\text {GeV}$. However, at the perturbative order considered in this study, such topologies arise only in QCD–EW interference terms that do not give rise to Breit–Wigner resonances. The dependence of our results on $\Gamma _t$ is thus completely negligible.

8 Here and in the following we adopt a power counting that does not include the extra factor $\alpha$ associated with vector-boson decays.

9 For what concerns process correlations, it is crucial that (apart from the $M_V$ dependence) all $V+$ jet processes are evaluated using equivalent dynamical scales.

10 In this regime, which is not the main focus of the present study, the process-correlation uncertainty (38) ceases to be a meaningful uncertainty estimate.

11 Note that, in spite of the fact that we present them as separate terms in Eq. (25), $\gamma$-induced contributions and NLO EW corrections to $pp\rightarrow V+$ jet are interconnected at $\mathcal {O}(\alpha ^2\alpha _{\mathrm {S}})$.

12 Here, in order to discuss qualitative features of Sudakov logarithms, we adopt a generic and rather schematic representation of the asymptotic high-energy limit. In particular, we do not consider some aspects, such as the helicity dependence of the corrections or SU(2) soft-correlation effects. However, in the numerical analysis all relevant aspects are consistently included.

13 See, e.g. Refs. [31, 79, 80] for a factorized treatment of QCD and EW corrections for Higgs-strahlung and vector-boson fusion processes.

14 As discussed below, the goodness of this naive Ansatz will be justified by fitting it to a realistic estimator of $\delta \kappa ^{(V)}_{{\mathrm {mix}}}(x)$.

15 In order to guarantee a proper cancellation of QCD and EW singularities, the jet algorithm is applied to all QCD partons and photons, excluding photons that are recombined with leptons, as well as the leading identified photon in case of the $\gamma +$jets process.

16 This is automatically achieved by reweighting MC samples generated with arbitrary PDFs with our complete $\mathrm {N}^k\mathrm {LO}\,{\mathrm {QCD}}\times \mathrm {nNLO}\,{\mathrm {EW}}$ predictions based on LUXqed_plus_PDF4LHC15_nnlo PDFs. Vice versa, restricting the reweighting to pure EW corrections and using MC samples based on different PDFs can lead to inconsistencies at $\mathcal {O}(\alpha )$.

17 Note that photon-induced contributions are not included in the summary plots of Fig. 17.
